# Task-irrelevant emotional expressions are not mimicked, but may modulate the mimicry of task-relevant emotional expressions

**DOI:** 10.3389/fpsyg.2024.1491832

**Published:** 2025-01-07

**Authors:** Heidi Mauersberger, Christophe Blaison, Ursula Hess

**Affiliations:** ^1^Department of Psychology, Humboldt-Universität zu Berlin, Berlin, Germany; ^2^Laboratoire de Psychologie Sociale, Université de Paris, Paris, France

**Keywords:** emotional mimicry, facial EMG, attention, task-relevance, affective priming

## Abstract

Emotional mimicry—the imitation of others’ emotions—is an empathic response that helps to navigate social interactions. Mimicry is absent when participants’ task does not involve engaging with the expressers’ emotions. This may be because task-irrelevant faces (i.e., faces that participants were instructed to ignore) are not processed. To assess whether processed task-irrelevant faces are also not mimicked, we conducted three studies [Study 1: *N* = 74 participants (27 men; *M_age_* = 26.9 years); Study 2: *N* = 53 participants (20 men; *M_age_* = 25.8 years); Study 3: *N* = 51 participants (7 men; *M_age_* = 26.8 years)] using an affective priming paradigm in which one face was task-relevant and one was to be ignored, as a framework to explore the impact of disregarded yet still perceptually processed faces on mimicry. We found that even though both faces were processed, only task-relevant faces were mimicked. Hence, our studies suggest that emotional mimicry depends not only on emotional processing *as such* but also on the way participants prioritize one piece of information over another. Further, task-irrelevant faces interfered with the mimicry of task-relevant faces. This suggests that even though incongruent task-irrelevant faces do not elicit an empathic (mimicry) response, they still may provide a context that can change the meaning of task-relevant faces and thus impact on the mimicry response.

## 1 Introduction

In everyday life, we are permanently surrounded by interaction partners who experience emotions: A friend who has just experienced a loss may express sadness or a colleague who has just won a prize may express pride and happiness. In all these instances, we are asked to react empathically to the emotions that have been expressed.

One important empathic reaction to others’ emotional expressions is emotional mimicry. Emotional mimicry—the imitation of the emotional signals of others—fosters mutual liking and strengthens social bonds (Fischer and Hess, 2017; Hess, 2021; Hess and Fischer, 2013). Even though emotional mimicry is an automatic, ballistic process, mimicry requires focal attention toward the emotional expression and its meaning. Specifically, the Mimicry as Social Regulator theory by Hess and Fischer (2013) proposes that people mimic not so much what they see but rather what they understand about an emotional expression within a given context and in alignment with their own goals. This implies that when observers do not pay attention to a given expressive stimulus, they would also not mimic this stimulus. This process could explain the limited mimicry response of emotional expressions that are deemed irrelevant for task completion (Seibt et al., 2015). Yet, affective information is generally processed automatically—even when individuals are told to ignore emotional facial expressions and shift their attentional focus elsewhere (Fazio et al., 1986). This implies that task-irrelevant emotional faces are probably processed in most cases. That is, lack of processing may not be the relevant factor to explain the lack of mimicry. Instead, we propose that the observers’ goals in the situation determine whether an expression will be mimicked or not. Yet, in all previous studies (see below), the processing of the (task-irrelevant) stimuli was not directly monitored. The present research aimed to determine if processed but task-irrelevant emotional expressions are mimicked. If they are not mimicked, this would further support the notion that mimicry can be modulated by observers’ goals—a view on mimicry that is still not agreed upon among all researchers in this domain (Niedenthal et al., 2005; Wood et al., 2016).

### 1.1 Emotional mimicry of task-relevant versus -irrelevant emotional expressions

A series of recent studies have provided strong evidence that emotional facial expressions (and body postures) elicit consistent and replicable behavioral effects, but crucially, these effects are observed *only* when the emotional content of the stimuli is *relevant* to the participant’s goals (Mirabella, 2018; Mancini et al., 2020, 2022; Calbi et al., 2022; Mirabella et al., 2022; Montalti and Mirabella, 2023, 2024). In these studies, participants completed two versions of a Go/No-go task in a counterbalanced order. In the emotional version of the Go/No-go task, participants were instructed to execute a motor response when presented with an emotional stimulus, such as a facial expression of anger, happiness, or fear, and to withhold their response when a neutral stimulus appeared on the screen (Mirabella, 2018; Mancini et al., 2020; Mirabella et al., 2022; Montalti and Mirabella, 2023, 2024). This setup was reversed in some instances, where participants responded to neutral stimuli and refrained from responding to emotional ones (Calbi et al., 2022; Mancini et al., 2022). The control task provided a critical contrast; it used the same set of images, but participants were required to focus on non-emotional aspects of the stimuli, such as the gender of the person in the image (Mirabella, 2018; Mancini et al., 2020, 2022; Mirabella et al., 2022; Montalti and Mirabella, 2023, 2024) or the color of their t-shirts (Calbi et al., 2022).

Across these studies, various experimental designs were employed, such as utilizing different body effectors (upper or lower limbs), presenting different emotional expressions (angry, happy, and fearful), and requiring different types of motor control (motor planning or motor inhibition). Despite these variations, the results consistently showed that emotional stimuli influenced behavioral responses only when they were directly relevant to the participants’ tasks. When the task required attention to the emotional content (as in the emotional version of the Go/No-go task), threatening stimuli like angry and fearful expressions garnered stronger attention and had a more significant effect on motor performance compared to happy expressions. Conversely, when the task required attention to non-emotional features (as in the control task), the emotional valence of the stimuli had no impact on participants’ responses. This body of research provides compelling evidence that the impact of emotional stimuli on behavior is intricately linked to the relevance of these emotions in the task context.

These findings support the goal-directed account proposed by Moors and Fischer (2019), which argues that all behaviors, including emotional behaviors, are driven by underlying goals. According to this view, the influence of observed emotional expressions on behavior is not automatic but depends on the relevance of the emotional content to the individual’s goals in a given context. In this vein, emotional mimicry—a facial response to others’ emotional expressions that signals emotional understanding and hence conveys empathy—is not just a reflexive reaction but a purposeful behavior shaped by context-specific goals. As such, the goal-directed approach predicts that mimicry is more likely to occur when observed expressions are relevant to the individual’s (social) objectives. People are more inclined to mimic others’ emotions when they serve their goals such as facilitating communication or fostering social cohesion. In fact, not all emotional expressions are mimicked equally. For example, mimicking a disgusted other may make less sense than mimicking a happy one—it is simply not beneficial to connect with someone who is signaling rejection (Hess and Fischer, 2013; Mauersberger et al., 2015, 2022b, 2022a).

In a narrower sense, emotional mimicry should only occur when others’ emotional expressions are directly relevant to the task at hand. The likelihood of mimicking another person’s emotion should increase when interpreting that emotion is crucial to the individual’s current goals or objectives. If the emotional expression is irrelevant to the task emotional mimicry should not take place.

**Figure 1:**
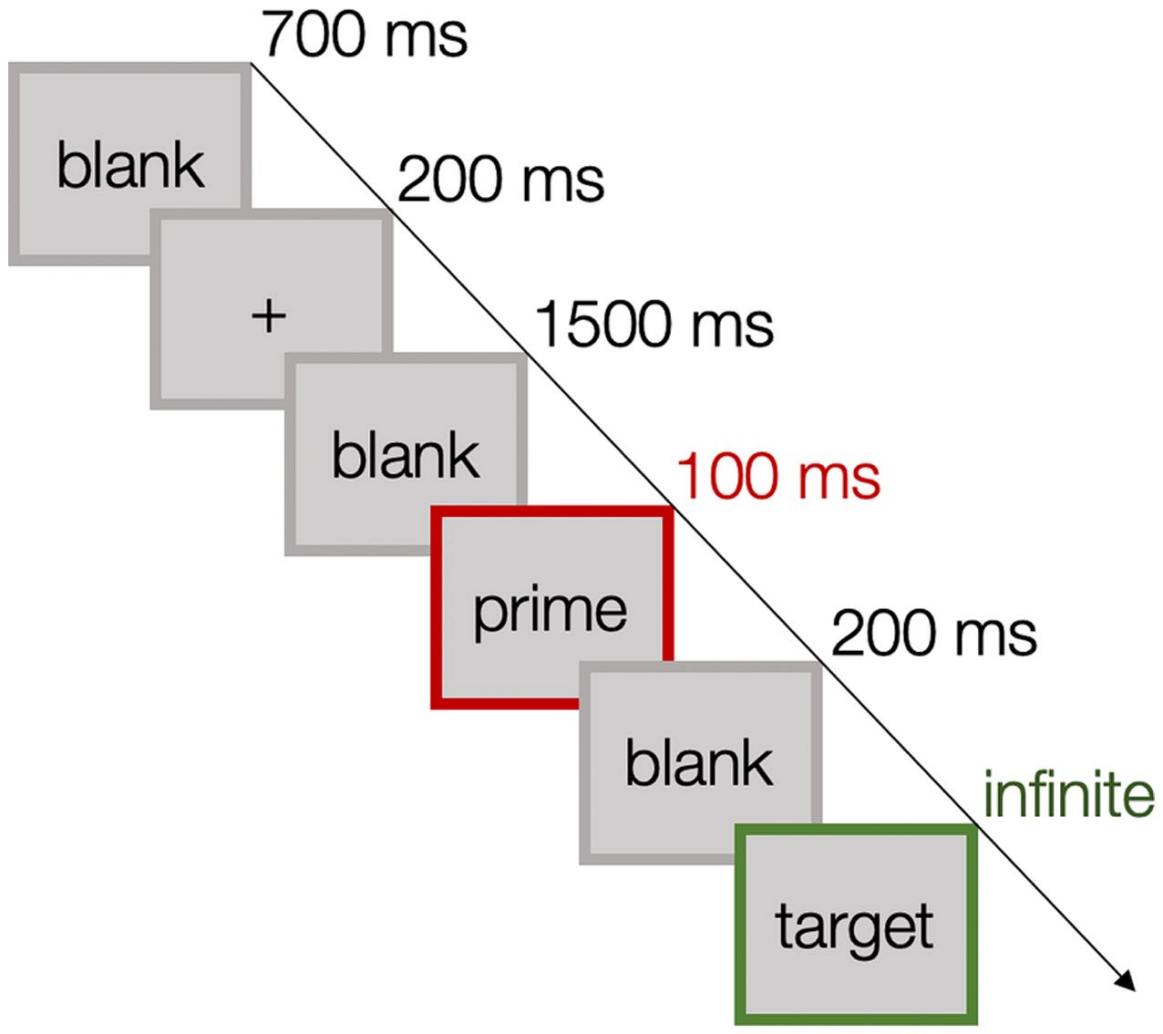
Elements and durations of one trial.

In fact, emotional mimicry can be absent when the task does not at least implicitly emphasize the relevance of the specific emotional content.^1^ For instance, Hess et al. (1998) instructed participants to judge to what extent a displayed emotion was genuine. Compared to other conditions, they found no mimicry, probably because the judgment did not require the decoding of the emotion. Similarly, Cannon et al. (2009) asked participants to categorize the color of blue-tinted or yellow-tinted depictions of angry and happy facial expressions. Figure 1 (right panel, Cannon et al., 2009, p. 926) shows a relative relaxation of the Corrugator Supercilii^2^ in response to angry target faces as well as no change from baseline for the Zygomaticus Major^3^ in response to happy target faces; these patterns are not congruent with a mimicry reaction. Indeed, even basic emotional processing phenomena such as the negativity bias (that is, individuals are slower in reacting to threatening compared to neutral stimuli) do not occur when individuals are asked to categorize emotional faces based on physical aspects (i.e., the color of a stimulus) for which the emotional content of the expression conveys no meaningful information (Van Dillen et al., 2011). In consequence, these emotional facial displays are then not mimicked (van Dillen et al., 2015). One explanation for these findings is that irrelevant expressions are not processed and thus are not mimicked.

In contrast, mimicry can be generally observed when individuals are asked to categorize emotional faces based on social dimensions (i.e., gender or personality of the expresser of the emotion) for which the emotional content of the expression may not be explicitly named but may still constitute a psychologically meaningful feature that is used for task completion. Specifically, facial expressions have a social signal value and they can inform about a person’s personality (Hareli and Hess, 2010). This may also explain the (at first sight) contradictory effects reported by Korb et al. (2014): In this study, participants were asked to rate the authenticity of smiles, which according to Hess et al. (1998) is a cognitive task that focuses the attention on non-affective aspects of the face thereby reducing the role of the emotional content of the facial expression. However, participants were given specific instructions that did not focus on facial features (i.e., such as explaining to participants that during genuine smiles wrinkles around the eyes occur; Frank et al., 1993) but rather on the underlying emotional meaning of the display (i.e., “‘the type of smile a person makes spontaneously when she is happy, joyful, or amused’” versus “‘the type of smile a person makes voluntarily when she wants to be polite, but *does not actually* feel very happy, joyful, or amused’”; Korb et al., 2014, p. 3). Hence, here also participants had to decode the emotional content of the display to complete the task, and thus emotional processing took place and mimicry occurred.

To sum up, mimicry typically occurs when tasks require individuals to make inferences about the expresser’s feelings. In contrast, when tasks do not require such inferences or when cognitive load is too high, mimicry does not occur, likely because the emotional information is not processed (van Dillen et al., 2015). This may also explain why the valence of emotional facial expressions does not significantly impact behavior in the non-emotional version of the Go/No-go task: The focus on non-emotional aspects may hinder the processing of emotional nuances, as the brain prioritizes task execution over emotional processing.

An alternate explanation is based on recent findings by Forbes et al. (2021). Specifically, these authors propose that self-relevance is necessary for the occurrence of mimicry. According to this view, only those emotional expressions are mimicked that are task- and thus self-relevant—that is, only those that are deemed meaningful in a certain context, as they help to fulfill a certain goal (such as accomplishing a task). This implies that task-irrelevant emotional expressions may be perceived and processed yet *not* mimicked. The present research had the goal to test this possibility.

One challenge in this context is to design a task that can confirm the processing of task-irrelevant emotional content without making that content task-relevant. Fortunately, the well-established (but still not infallible, see, Bodenschatz et al., 2019) affective priming paradigm specifically allows monitoring stimulus processing while—at the same time—rendering some stimuli irrelevant and directing the focus elsewhere (see Fazio, 2001). For this task, individuals have to categorize the valence of a target, which is presented shortly after a briefly presented prime. As judging the target’s valence takes longer when the prime’s valence is incongruent (instead of congruent), we know that the prime is processed even though it is not relevant to the decision-making process.

We therefore used an adapted affective priming paradigm to assess the mimicry of task-irrelevant expressions. In our studies, both primes and targets were emotional facial expressions (to allow mimicry of the target as well as of the prime; further see Beall and Herbert, 2008, for the finding that emotional displays produce stronger interference effects than positive and negative words) and we also included neutral expressions as both primes and targets (see below for our rationale behind that choice).

### 1.2 Briefly presented faces as contexts that influence emotional mimicry

Mimicry serves an affiliation goal. Hence, expressions displayed by disliked others, members of an out-group, or individuals with whom we compete are mimicked to a lesser degree or not at all (see Hess and Fischer, 2013, for a review) because they do not entrain affiliation. Yet, the information gleaned from briefly presented primes may also suffice to affect affiliation goals and thereby modulate mimicry responses. Specifically, there is evidence that automatic evaluations of subliminally presented stimuli influence deliberate judgments of following stimuli (Ferguson et al., 2005). In fact, subliminally presented angry and happy facial expressions influence how likable and warm a person is perceived to be (Anderson et al., 2012). As such, even though one may correctly perceive the joy in a target face, this happy face may not transmit the same warmth and affiliativeness if preceded by an angry face than if preceded by a happy face. Thus, faces that follow each other can potentially function as a context for each other and in this sense also affect mimicry. Consequently, it is plausible that task-irrelevant expressions can still affect mimicry to task-relevant expressions, even if they are not mimicked themselves.

### 1.3 The present studies

As mentioned above, the present studies are based on an affective priming paradigm (see Fazio, 2001) as a scaffold to investigate the effects of ignored but nevertheless at least perceptually processed faces on mimicry. In a classic semantic priming paradigm, participants first see either a positive or negative emotional stimulus, which is very briefly presented—the *prime,* and then see a second emotional stimulus that is also either positive or negative in valence, which is presented until participants press a button to indicate the valence of this second item—the *target*. This results in a relatively faster response when prime and target are congruent in valence rather than incongruent.

We changed the task relevance of the prime across three studies by changing participants’ instructions for the affective priming task. Whereas participants were told to ignore the prime (in our case a facial expression) and categorize the target (also a facial expression) in Study 1 (classic design), they were told to pay attention to the prime in Study 2 (but still respond to the target). This adjustment (regard versus disregard the prime) assures that participants pay attention to the prime. However, the prime is still task-irrelevant, as it does not help to identify the target’s valence (to the contrary, it interferes with the rating of the target in 50% of the time when its valence is incongruent with the valence of the target). Hence, we did not expect that this manipulation would lead to an increase in task- (or self-) relevance and thus to a different pattern in the mimicry response. However, it serves to show that attention alone is not the same as task relevance.

In Study 3, we increased the task- (or self-) relevance of the prime and decreased the task- (or self-) relevance of the target by instructing participants to categorize the prime and ignore the target. Consequently, we expected an opposite pattern of results concerning the mimicry response. Primes should be mimicked and targets should not be mimicked.

To adapt the design for the purpose of assessing mimicry, we included neutral faces and we used expressions by the same expresser for primes and targets. Due to these changes, affective priming effects may not necessarily point to semantic processing of the prime but rather are suggestive of perceptual priming. Yet, the precise nature of the priming effect holds limited relevance in the current context, given that the primary aim of assessing the priming effect was to confirm participants’ processing of the primes. Importantly, since most neutral faces do convey some affective meaning due to the morphology of the face (for example, low eyebrows seem more negative, and highly curved ones more positive; Adams et al., 2012), it is still possible to complete the affective priming task in the trials where individuals need to react to neutral targets or primes. However, since there is no expression to mimic nor a clear emotional signal to understand, it is unlikely that these expressions result in mimicry. In fact, in studies that include neutral faces, no facial reactions to these faces are typically observed (see, e.g., Kastendieck et al., 2021).

We measured mimicry with facial electromyography (EMG). Facial EMG measures the electrical signals generated by facial muscles in the process of producing facial expressions. Hence, facial EMG is a valid measure of emotional facial expressions (Girard et al., 1997) that has a high spatial resolution (Tassinary et al., 2007). Importantly, facial EMG can detect even subtle facial movements such as facial mimicry reactions (Hess et al., 2017).

The addition of “neutral” primes and targets made it possible to measure the full course of mimicry to both primes and targets as mimicry responses are relatively slow and only peak at 1 s after stimulus presentation. This means that responses to primes and targets may overlap. In other words, emotional mimicry responses to the target (Studies 1 and 2) or prime (Study 3) could interfere with or overshadow mimicry responses to the prime (Studies 1 and 2) or target (Study 3) and neutral targets (Studies 1 and 2) and primes (Study 3) were used to disentangle these effects.

In sum, our studies aimed to explore whether mimicry hinges on task relevance and cognitive engagement with the goal to better understand the dynamics of empathy and social connectedness in diverse contexts, from friendships to workplace interactions to broader social networks.

### 1.4 Hypotheses

For all three studies, we hypothesized that mimicry responses only occur in response to task-relevant faces. In Studies 1 and 2, targets were task-relevant and primes were task-irrelevant. By contrast, in Study 3, targets were task-irrelevant and primes were task-relevant. We further predicted that task-irrelevant expressions, even when not mimicked, can nonetheless serve as context for the task-relevant expressions that thereby influence the timing and degree of mimicry of task-relevant expressions.

## 2 Study 1

Study 1 used classic affective priming instructions where participants are told to ignore the prime and to judge the target for valence. Thus, the prime is task-irrelevant and hence should not be mimicked, whereas the target is task-relevant and hence should be mimicked. Still, happy primes may interfere (and hence should reduce) the mimicry of angry targets and vice versa.

It was not our intention for the primes to be subliminal; therefore, we selected a presentation duration of 100 ms. This duration is sufficient for the primes to be consciously perceived by participants. Further, even 100 ms is ample to allow for mimicry reactions, which can even be found for presentation times as short as 17 ms (Sonnby-Borgström, 2002).

### 2.1 Methods

#### 2.1.1 Participants

In typical mimicry experiments in our laboratory, effect sizes tend to range from $\eta_{p}^{2}$ = 0.15 to $\eta_{p}^{2}$ = 0.3. Classic studies by Dimberg (1982) even found effect sizes as high as $\eta_{p}^{2}$ = 0.51 for the focal muscle site by emotion interaction. Using the conservative effect size of $\eta_{p}^{2}$ = 0.15 and aiming for 90% power at alpha = 0.05, 51 participants would be required. Given that the current design is relatively complex, we decided to oversample by using a sample size commensurate with research on affective priming.

We recruited a total of 74 participants (27 men) with a mean age of 26.9 years (*SD* = 6.78 years) via the participant acquisition server at Humboldt-University. They participated individually and received either course credit (psychology students) or €8 for their participation.

Participants were aware that they had the right to discontinue participation at any time, that their responses were confidential, and that collected data would be stored in a pseudonymized way according to the European General Data Protection Regulation (GDPR). All three studies were approved by the Institutional Ethics committee (Ethics Committee of the Institute of Psychology at Humboldt-University; Application 2013–16) and were carried out in accordance with the guidelines of the Declaration of Helsinki (except for lack of preregistration), and the recommendations for good scientific practice of the Deutsche Forschungsgemeinschaft (German Research Foundation, DFG). The data collection (for all three studies) took place from April 16, 2013 to October 01, 2014.

#### 2.1.2 Procedure

Upon arrival at the laboratory, each participant was greeted by the experimenter and seated in a comfortable chair in front of a computer. Then they were informed that their task would be to rate a series of facial expressions regarding the emotional expression displayed. Participants who gave written informed consent (outlining the study’s purpose, procedures, risks, and benefits) received detailed instructions regarding the task (“you will see two facial expressions, which will be presented one after the other with a brief delay; please ignore the first one and only focus on and categorize the emotion of the second one”) and the experimenter attached the electrodes.

Prior to the experimental task, participants watched a relaxing baseline video. Participants then completed 10 practice trials. Each trial began with a fixation cross (200 ms), followed by a blank screen (1,500 ms), which was replaced by the prime (100 ms), followed again by a blank screen (200 ms), and then the target appeared and remained until response. Participants pressed the P or the Q-key to indicate that the target showed either a positive or negative emotional expression. We recorded response times from the onset of the target. Response-key assignment was counterbalanced across participants. After the response and a subsequent inter-trial interval (700 ms), the next trial was presented (see Figure 1 for a visualization of the elements and durations of a trial).

Following the practice trials, the experimenter verified that participants understood the task and had no further questions. Participants then completed the test phase consisting of 8 blocks of 40 trials each, with a short pause between blocks. Across every two blocks, there was an equal number of each prime-target pair type (i.e., 10 happy prime/happy target, 10 happy prime/angry target, 10 happy prime/neutral target, 10 angry prime/angry target, 10 angry prime/happy target, 10 angry prime/neutral target, 10 neutral prime/happy target, 10 neutral prime/angry target); within each of the 10 pairs of each type, there were five for each sex. Prime and target actors within each trial were always the same. The same combination of stimuli was not shown twice in a row. At the end of the experiment, participants were fully debriefed,^4^ and all outstanding questions were answered by the experimenter.

#### 2.1.3 Stimulus material

Stimuli were taken from a set of standardized photographs—the Radboud Faces Database (RaFD; Langner et al., 2010)—which provides facial expressions of different emotions presented by different actors. Emotional facial expressions were based on the movement of specific facial action units as described in the Facial Action Coding System (Ekman et al., 2002). Expressions from 10 male and 10 female actors were selected for use in the present studies.^5^ For the purpose of our studies, we used static frontal photographs with forward-facing gaze and angry, happy, and neutral expressions. The expressions in the set are well recognizable: Happy - 98%, angry - 85%, neutral - 84%.

#### 2.1.4 Dependent measures

*Reaction times.* Trials with reaction times (RTs) <150 ms were excluded from analyses (0.01%), as they are too fast for a standard visuomotor response and more likely the result of anticipatory or accidental button pressing. We further excluded trials with incorrect responses (4.8%) or with RTs above 1,500 ms (2.7%) from analyses (see, e.g., De Houwer and Randell, 2004).^6^ It is important to note that even though neutral faces carry some affective meaning (Adams et al., 2012), and the task is therefore not impossible, this nonetheless represents a different task. We therefore did not include trials with neutral targets in the *main* analyses.

*Facial muscle activity.* Activity over the *Zygomaticus Major, Orbicularis Oculi,* and *Corrugator Supercilii* regions was recorded with facial electromyography (EMG) on the left side of the face using bipolar placements of 13/7 mm Ag/AgCl surface-electrodes according to the guidelines established by Fridlund and Cacioppo (1986). The EMG raw signal was sampled at 1000 Hz using a MindWare Technologies BioNex Bio-Potential Amplifier. Raw data were filtered with a 30–300 Hz bandfilter and a 50 Hz notch filter and rectified and smoothed with a 5 Hz low-pass filter. The data were manually checked for artifacts based on predefined criteria such as coughing or sneezing. Segments containing artifacts were removed and the data were aggregated into 100 ms bins. Then, baseline^7^ to trial difference scores were calculated and within-subject z-transformed.

For data analysis, a pattern score was calculated as described by Hess et al. (2017), which indicates the contrast between the average activity of Zygomaticus M. and O. Oculi minus the activity of Corrugator S. Thus, happiness mimicry is indexed by a positive pattern score (that is, a pattern score above zero) and anger mimicry by a negative pattern score (that is, a pattern score below zero). That means that *target* mimicry takes place when during trials with happy targets, participants’ facial reactions are positive and during trials with angry targets, participants’ facial reactions are negative. Similarly, *prime* mimicry takes place when during trials with happy primes, participants’ facial reactions are positive, and during trials with angry primes, participants’ facial reactions are negative. Accordingly, if, for instance, facial reactions are less positive during trials with angry primes and happy targets than during trials with happy primes and happy targets, but they are still positive or about zero (and not negative), it would mean that primes impair target mimicry but not that primes as such are mimicked. Further, the 100 ms bins were aggregated into four distinct time segments: Prime (0–300 ms), target onset (300–600 ms), target evaluation (600–1000s), and blank screen (1000–1,400 ms).

#### 2.1.5 Data analysis strategy

To analyze our data on a trial-based level, we conducted linear mixed model (LMM)^8^ (Brown, 2021) analyses with the *lmerTest* (Kuznetsova et al., 2017) extension for *lme4* (Bates et al., 2014) with the fixed factors target emotion (happy, angry) and prime emotion (happy, angry, neutral) on RTs to investigate the processing of the task-irrelevant (disregarded) faces and with the fixed factors target emotion (happy, angry), prime emotion (happy, angry, neutral) and segment (prime, target onset, target evaluation, blank screen) on facial responses to investigate the mimicry responses. We included all factors in the analysis. This approach is aimed at a structured testing of our theoretical framework, where each factor in the model serves a specific theoretical purpose.

Participant ID was used as a cluster variable with random intercept; random slopes were not specified due to non-convergence. If significant (two-way) interactions emerged, we calculated simple slopes as post-hoc tests of the relationships between the (two) predictors and the outcome variables. Simple slopes are separate regression lines for different levels of one predictor variable (that is, the moderator) to see how the relationship between the other predictor variable and the outcome variable changes at different levels of the moderator. We report the main analyses below, for further analyses please find our data and the corresponding codebook, at: https://osf.io/r3vk4/?view_only=dd37e69c8e5b44ac97259ee179e2a72f.

### 2.2 Results and discussion

#### 2.2.1 Reaction times

**Figure 2:**
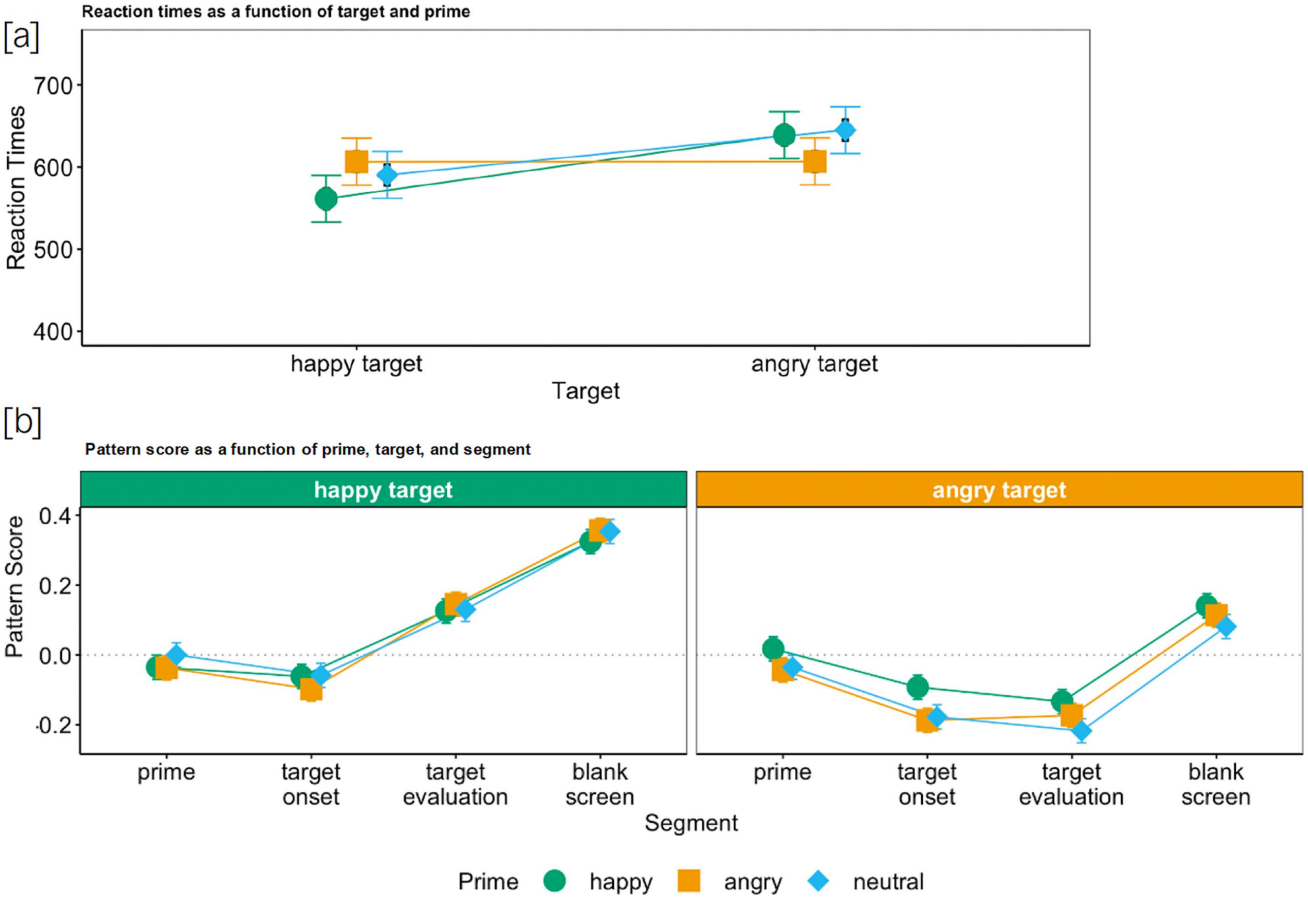
Reaction times **(a)** and pattern score **(b)** as a function of target, prime (and segment) for Study 1. Colored bars represent 95% confidence intervals and black bars represent standard errors.

We first analyzed whether task-irrelevant (disregarded) faces (i.e., the primes) were processed. The LMM analysis on RTs revealed a significant main effect of target emotion, *semi-partial R^2^* = 0.012,^9^ (Nakagawa and Schielzeth, 2013; Jaeger et al., 2017) and a significant main effect of prime emotion, *semi-partial R^2^* = 0.001, which were qualified by an interaction between prime emotion and target emotion, *semi-partial R^2^* = 0.006: As expected (Figure 2a), the RTs were significantly shorter when target and prime emotion matched than for incongruent combinations, *b*_ang-hap_ang-hap_ = −77.1, *t* = −12.3, *p* < 0.001, *CI*_95_ ang-hap_ang-hap_ = [−89.4, −64.8]. These findings confirm that primes were processed despite being irrelevant to the task (also see Supplementary Table S2, which displays all coefficients for the analysis and reports the corresponding simple slopes).^10^

#### 2.2.2 Facial muscle activity

Then, we investigated whether only task-relevant faces (the targets) were mimicked. The LMM on the facial responses revealed a main effect of target emotion, *semi-partial R^2^* = 0.006, *b*_ang-hap_ = −0.15, *t* = −19.3, *p* < 0.001, *CI*_95_ang-hap_ = [−0.17, −0.14]. Overall, facial reactions in response to a happy target were positive, *M*_hap_ = 0.10, *CI*_95_hap_ = [0.08, 0.11], whereas they were negative in response to an angry target, *M*_ang_ = −0.06, *CI*_95_ang_ = [−0.07, −0.04] (Figure 2b) confirming both happy and angry target mimicry.

Further, there was a main effect of prime emotion, *semi-partial R^2^* = 0.0001, which was qualified by an interaction between target emotion and prime emotion, *semi-partial R^2^* = 0.0003, *b*_ang-hap*ang-hap_ = −0.06, *t* = −3.00, *p* = 0.003, *CI*_95_ang-hap*ang-hap_ = [−0.10, −0.02]. Specifically, for angry targets, the facial expression was less negative when the prime was incongruent (happy instead of angry), simple slope *z*_ang_ang-hap_ = −0.06, *t* = −4.00, *p* < 0.001, *CI*_95_ang_ang-hap_ = [−0.08, −0.03] (Figure 2b). No such effect was found for happy targets. Hence, facial reactions in response to a happy target were always positive (independent of the preceding prime), whereas they were only negative in response to an angry target when the prime was also angry, *M*_ang_ang_ = −0.07, *CI*_95_ang_ang_ = [−0.09, −0.05], or when it was neutral, *M*_ang_neu_ = −0.09, *CI*_95_ang_neu_ = [−0.11, −0.07], but not when it was happy, *M*_ang_hap_ = −0.02, *CI*_95_ang_hap_ = [−0.04, 0.004]. Consequently, mimicry of angry targets was reduced when preceded by incongruent (happy) primes, but no such effect was observed for happy targets. Thus, participants overall mimicked the target rather than the prime expressions.

Furthermore, the time course shows that participants’ facial responses to all target expressions showed a negative response ca. 100–300 ms after prime onset (see Table 1). This brief reaction, however, despite an onset prior to target presentation, was more pronounced for angry targets than for happy targets (see Table 2). Further, it was solely driven by an increase in Corrugator S. activity (see Supplementary Figure S1). Given the timing and restriction to Corrugator S. (see also, Dimberg and Thunberg, 1998), this brief facial reaction can be best explained as an orienting response to the prime (Hess et al., 2016).

**Table 1:** Time course of participants’ facial reactions (Study 1).

Predictors	Estimates	*t*	95% CI	*p*
Segment (Target Onset–Prime)	−0.09	−7.96	[−0.11, −0.07]	**<0.001**
Segment (Target Onset–Prime) * Target (Angry–Happy)	−0.08	−3.69	[−0.13, −0.04]	**<0.001**
Segment (Target Evaluation–Target Onset)	0.09	8.13	[0.07, 0.11]	**<0.001**
Segment (Target Evaluation–Target Onset) * Target (Angry–Happy)	−0.23	−10.09	[−0.27, −0.18]	**<0.001**
Segment (Blank Screen–Target Evaluation)	0.25	22.0	[0.23, 0.27]	**<0.001**
Segment (Blank Screen–Target Evaluation) * Target (Angry–Happy)	0.08	3.34	[0.03, 0.12]	**<0.001**

Bold values indicate *p*-values below 0.05, indicating statistical significance.

**Table 2:** Simple slopes for the interaction of segment and target on participants’ facial reactions (Study 1).

Predictors	Estimates	*t*	95% CI	*p*
Segment (Target Onset–Prime) for Angry Target	−0.13	−8.24	[−0.16, −0.10]	**<0.001**
Segment (Target Onset–Prime) for Happy Target	−0.05	−3.02	[−0.08, −0.02]	**<0.001**
Segment (Target Evaluation–Target Onset) for Angry Target	−0.02	−1.39	[−0.05, 0.01]	0.16
Segment (Target Evaluation–Target Onset) for Happy Target	0.21	12.9	[0.17, 0.24]	**<0.001**
Segment (Blank Screen–Target Evaluation) for Angry Target	0.29	17.9	[0.26, 0.32]	**<0.001**
Segment (Blank Screen–Target Evaluation) for Happy Target	0.21	13.2	[0.18, 0.24]	**<0.001**

Bold values indicate *p*-values below 0.05, indicating statistical significance.

From target onset to target evaluation, the largest difference between angry targets and happy targets could be observed (see Table 1). That is, facial responses became more positive for happy targets and did not change for angry targets (see Table 2), indicative of happiness as well as anger mimicry (of the target). In the end, facial responses became overall (more) positive (from target evaluation to blank screen, see Table 1) and this final change was more pronounced for angry targets than for happy targets (see Table 2; Supplementary Table S2, which displays all coefficients of the analysis). Thus, irrespective of the valence of the target, participants smiled in the end (after they had made their decision about the valence of the target). Yet, this smile was more subtle following an angry than following a happy target.

**Figure 3:**
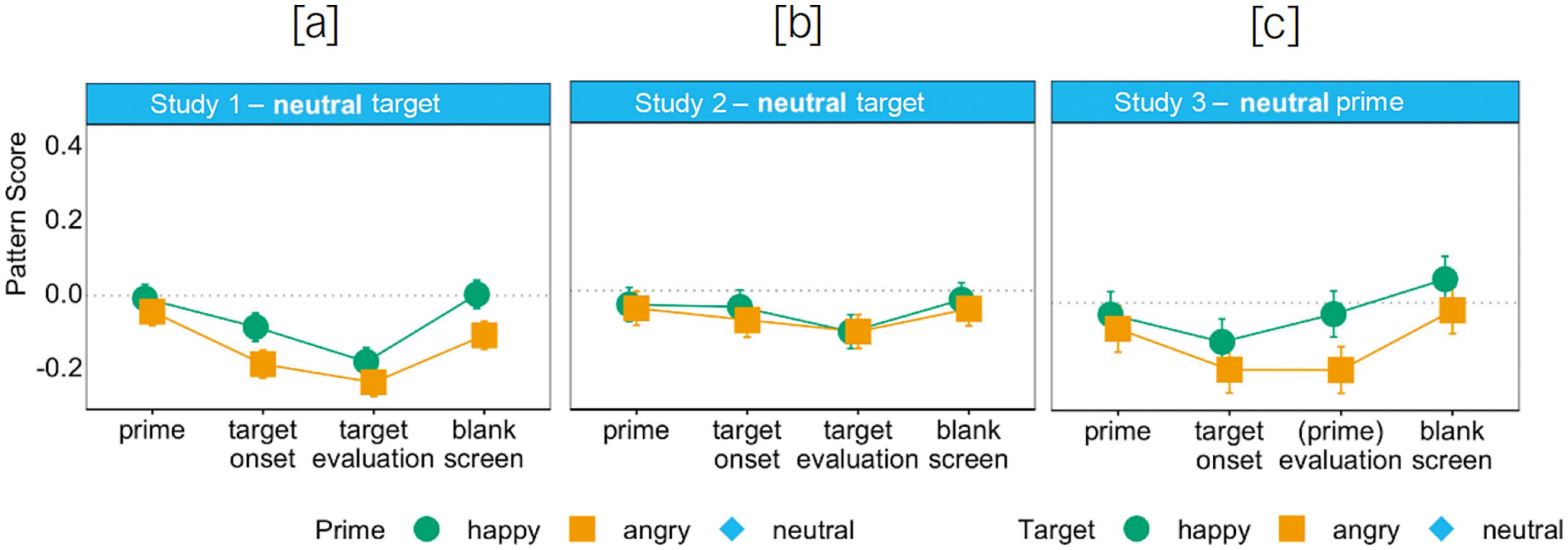
Pattern score as a function of prime and segment (Studies 1 and 2) and target and segment (Study 3) for neutral targets in Studies 1 **(a)** and 2 **(b)** and neutral primes in Study 3 **(c)**.

*Facial reactions to neutral targets.* To address the concern that the mimicry of targets interrupted the mimicry of primes, we conducted *additional* analyses to assess prime mimicry for the neutral targets. As they show no specific emotion, “mimicry” of neutral targets cannot interfere with facial muscle activity due to prime mimicry. In fact, in studies where neutral expressions are included, no facial reactions to such expressions are observed (e.g., Kastendieck et al., 2021). For this, we conducted an LMM analysis with the fixed factors prime emotion (happy, angry) and segment (prime, target onset, target evaluation, blank screen) on facial responses to the neutral target. Here again, we can observe that even though facial reactions were initially more positive during happy primes than during angry primes, *b*_ang-hap_ = −0.07, *t* = −4.73, *p* < 0.001, *CI*_95_ang-hap_ = [−0.09, −0.04], no prime mimicry occurred, as expressions during neutral targets were overall negative for both happy primes, *M*_hap_ = −0.07, *CI*_95_hap_ = [−0.10, −0.14], and angry primes, *M*_ang_ = −0.14, *CI*_95_ang_ = [−0.17, −0.11] (see Figure 3a; Supplementary Table S5, which displays all coefficients of the analysis). Hence, participants did not mimic the prime.

#### 2.2.3 Task-irrelevant faces were processed but not mimicked

Our findings support our hypothesis that—even though task-irrelevant faces are processed—they are not mimicked. Thus, a lack of emotional processing (Seibt et al., 2015) cannot explain why mimicry is reduced when individuals do not focus on the emotional content of a facial expression. Rather, mimicry was driven by the task relevance of the image, that is, by the degree that it contributed to participants’ goals in the experiment. This suggests that top-down cognitive processes, specifically participants’ task goals, are able to suppress automatic responses (i.e., emotional mimicry) to an emotional stimulus that has been visually perceived and processed at a very early stage. This contradicts the notion that emotional stimuli automatically trigger motoric and somatic responses and controlled processes interact with these responses only afterwards (see, e.g., Heyes, 2018). In fact, this finding aligns with recent claims that challenge simplistic views of responses to emotional stimuli and emphasize the role of emotions in complex, adaptive behavior (Moors and Fischer, 2019).

#### 2.2.4 Task-irrelevant faces influenced the level of mimicry of task-relevant faces

Nevertheless, task-irrelevant incongruent primes interfered with the mimicry of target faces by slightly reducing the overall effect, especially for angry targets. Since the overall pattern was strongly indicative of target mimicry, this finding suggests that incongruent primes may serve as a context that changes the meaning of the angry target. This finding is also in line with findings by Philip et al. (2018) who used an affective priming paradigm using word primes and who found that positive word primes suppressed the mimicry of angry targets, whereas negative word primes did not influence the mimicry of happy targets.

## 3 Study 2

Study 2 was a replication of Study 1, except that we asked participants to focus their attention on the prime. Still, they had to categorize the target’s valence and hence, even though the prime was given more attention, it was still irrelevant to the decision-making process. This manipulation addresses the question of whether attention alone suffices to induce mimicry. We did not hypothesize this to be the case and therefore expected a similar pattern of results as in Study 1.

### 3.1 Methods

The procedure, the stimulus material, and the dependent measures were the same for Study 2 as for Study 1. The only difference was the specific instructions regarding the task participants received (“you will see two facial expressions, which will be presented one after the other with a brief delay; please focus on the first one and categorize the emotions of the second one”). Here also, trials with neutral targets, incorrect responses (4.1%), or with response times (RTs) below 150 ms (0.12%) or above 1,500 ms (5.6%) were excluded from the main analyses.^11^

#### 3.1.1 Participants

Based on the results of Study 1, we used the *simr* package (Green and Macleod, 2016) to run a simulation-based LMM power analysis. That is, we used the smallest significant effect of *b*_ang-hap*ang-hap_ = −0.06 (that is, the effect of the interaction between target and prime) of the analysis predicting facial reactions to calculate the minimum sample size for Study 2 (to be able to achieve at least 80% power at an alpha level of 0.05). The power analysis pointed to recruiting about 40 to 50 participants (see power curve in Supplementary Figure S2).

Hence, a total of 53 participants (20 men) with a mean age of 25.8 years (*SD* = 4.30 years) were recruited via the participant acquisition server at Humboldt-University. Again, participants were paid €8 or received course credit.

### 3.2 Results and discussion

#### 3.2.1 Reaction times

**Figure 4:**
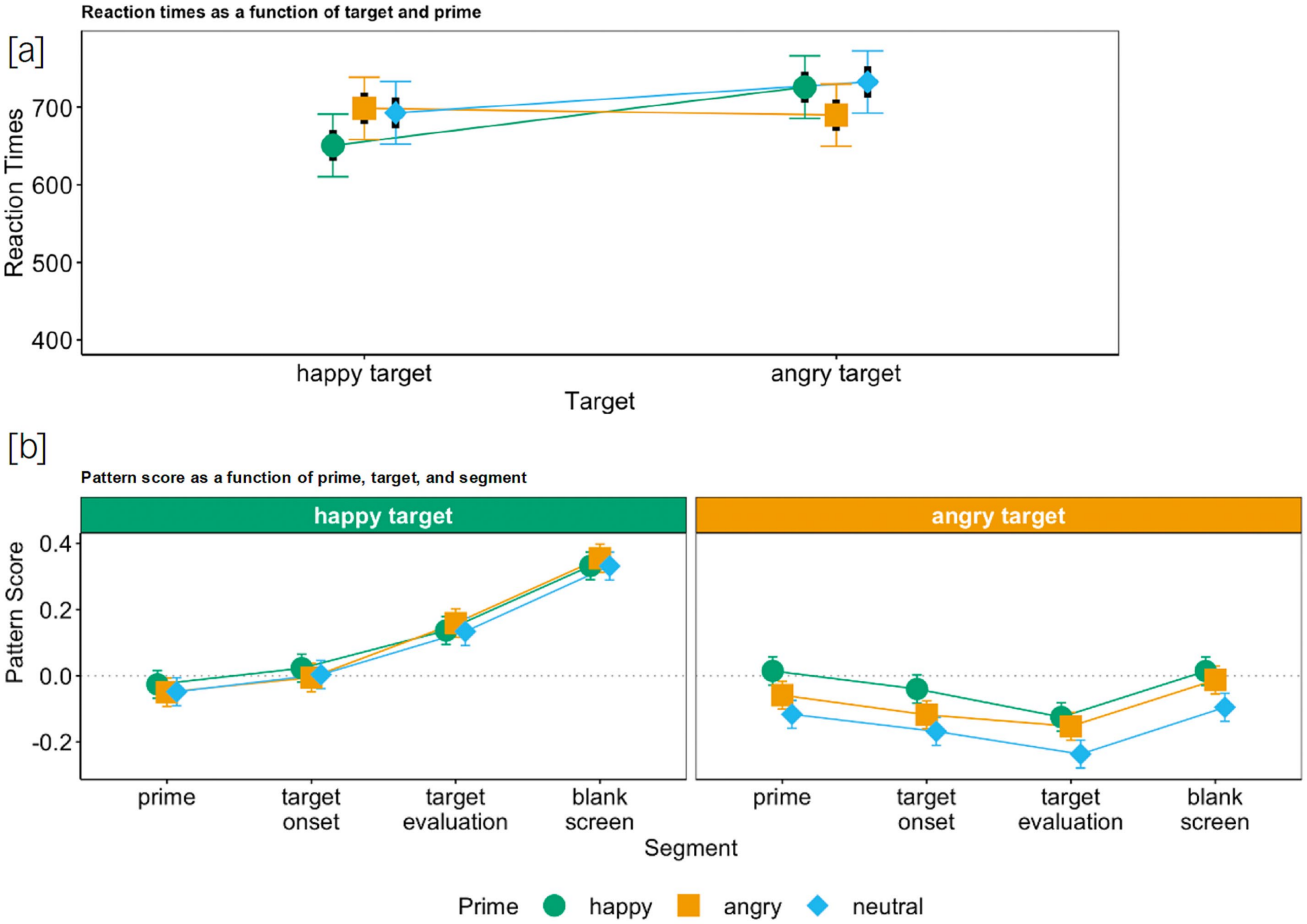
Reaction times **(a)** and pattern score **(b)** as a function of target, prime (and segment) for Study 2. Colored bars represent 95% confidence intervals and black bars represent standard errors.

Similar to Study 1, we found a significant main effect of target emotion, *semi-partial R^2^* = 0.005, and a significant main effect of prime emotion, *semi-partial R^2^* = 0.002, which, however, again were qualified by an interaction between prime emotion and target emotion, *semi-partial R^2^* = 0.005: As expected (Figure 4a), the RTs, here also, were significantly shorter when target and prime emotion matched than for incongruent combinations, *b*_ang-hap_ang-hap_ = −84.0, *t* = −9.18, *p* < 0.001, *CI*_95_ ang-hap_ang-hap_ = [−102.0, −66.1], confirming again that primes were processed despite their irrelevance to the decision-making task (also see Supplementary Table S7, which displays all coefficients of the analysis and the corresponding simple slopes).^12^

#### 3.2.2 Facial muscle activity

As in Study 1, we found a main effect of target emotion, *semi-partial R^2^* = 0.009, *b*_ang-hap_ = −0.20, *t* = −20.0, *p* < 0.001, *CI*_95_ang-hap_ = [−0.22, −0.18]. Overall, facial reactions in response to a happy target were positive, *M*_hap_ = 0.11, *CI*_95_hap_ = [0.10, 0.13], whereas they were negative in response to an angry target, *M*_ang_ = −0.09, *CI*_95_ang_ = [−0.11, −0.08] (Figure 4b), which means that both happy as well as angry targets were mimicked.

Further, just as in Study 1, incongruent primes slightly reduced mimicry of angry targets; nevertheless, participants *only* mimicked the task-relevant targets. That is, the main effect of prime emotion, *semi-partial R^2^* = 0.0006, was qualified by an interaction between target emotion and prime emotion, *semi-partial R^2^* = 0.0004, *b*_ang-hap*ang-hap_ = −0.05, *t* = −2.00, *p* = 0.045, *CI*_95_ang-hap*ang-hap_ = [−0.10, −0.001]. That is, for angry targets, the facial expression was less negative when the prime was incongruent (looked happy instead of angry), simple slope *z*_ang_ang-hap_ = −0.05, *t* = −2.93, *p* = 0.003, *CI*_95_ang_ang-hap_ = [−0.09, −0.02] (Figure 4b). Nonetheless, facial reactions in response to an angry target were always negative, indicative of target mimicry. Similar to Study 1, angry primes did not affect the mimicry of happy faces.

Furthermore, the time course shows that from prime to target onset, an initial increase in Corrugator S. activity (the same orienting response as in Study 1, which however was somewhat less pronounced here) was followed by a decrease in Corrugator S. activity during happy targets and a further increase during angry targets (see Supplementary Figure S3). Then, the positive change in participants’ facial responses from target onset to target evaluation was qualified by an interaction of segment and target emotion (see Table 3). That is, facial responses became more positive for happy targets and more negative for angry targets (see Table 4). Hence, exactly as in Study 1, from target onset to target evaluation, we can clearly observe happiness as well as anger mimicry (of the target). In the end (from target evaluation to blank screen), facial responses became overall (more) positive (and this final change was less pronounced for angry targets than for happy targets, see Table 3; Supplementary Table S8, which displays all coefficients of the analysis). Thus, similar to Study 1, irrespective of the valence of the target, participants smiled in the end (after they had made their decision about the valence of the target). Yet, this smile was more subtle after an angry target than after a happy target.

**Table 3:** Time course of participants’ facial reactions (Study 2).

Predictors	Estimates	*t*	95% CI	*p*
Segment (Target Onset–Prime)	−0.001	−0.24	[−0.03, 0.02]	0.81
Segment (Target Onset–Prime) * Target (Angry–Happy)	−0.10	−3.61	[−0.16, −0.05]	**<0.001**
Segment (Target Evaluation–Target Onset)	0.04	2.56	[0.01, 0.06]	**0.011**
Segment (Target Evaluation–Target Onset) * Target (Angry–Happy)	−0.20	−6.93	[−0.23, −0.14]	**<0.001**
Segment (Blank Screen–Target Evaluation)	0.17	11.7	[0.14, 0.20]	**<0.001**
Segment (Blank Screen–Target Evaluation) * Target (Angry–Happy)	−0.06	−1.96	[−0.11, 0.00]	**0.049**

Bold values indicate *p*-values below 0.05, indicating statistical significance.

**Table 4:** Simple slopes for the interaction of segment and target on participants’ facial reactions (Study 2).

Predictors	Estimates	t	95% CI	*p*
Segment (Target Onset–Prime) for Angry Target	−0.06	−2.72	[−0.09, −0.02]	**0.006**
Segment (Target Onset–Prime) for Happy Target	0.05	2.38	[0.01, 0.09]	**0.012**
Segment (Target Evaluation–Target Onset) for Angry Target	−0.06	−3.09	[−0.10, −0.02]	**0.002**
Segment (Target Evaluation–Target Onset) for Happy Target	0.14	6.70	[0.10, 0.18]	**<0.001**
Segment (Blank Screen–Target Evaluation) for Angry Target	0.14	6.91	[0.10, 0.18]	**<0.001**
Segment (Blank Screen–Target Evaluation) for Happy Target	0.19	9.67	[0.16, 0.24]	**<0.001**

Bold values indicate *p*-values below 0.05, indicating statistical significance.

*Facial reactions to neutral targets.* In this analysis, we could not find an effect of prime emotion (see Figure 3b; Supplementary Table S10, which displays all coefficients of the analysis). Hence, participants did not mimic the prime.

In sum, replicating Study 1, Study 2 confirmed that task-irrelevant primes were processed and interfered with the target mimicry but clearly were *not* mimicked, even when participants were instructed to focus on them. This finding supports the idea that mimicry is driven by the task relevance of facial expressions.

## 4 Study 3

Study 3 was a replication of Study 1 and Study 2, except that we asked participants to focus their attention on the prime and categorize the valence of the prime while disregarding the target. That is, functionally targets and primes changed places and prime valence now became the focus of participants’ attention. Hence, we expected that participants would mimic the prime, as the prime was task-relevant whereas the target was not. Thus, we expected the converse pattern of results found in Studies 1 and 2. Since primes were still shown prior to the target, we expected a backward affective priming effect (see Fockenberg et al., 2006, for the finding that backward affective priming works almost as well as forward affective priming).^13^

Importantly, as primes were still only shown briefly (100 ms), whereas targets were shown until a button was pressed, it is not implausible that incongruent targets may interfere more with both the ratings and the mimicry of primes than primes interfered with the ratings and mimicry of targets in Studies 1 and 2.

### 4.1 Methods

The procedure, the stimulus material, and the dependent measures were the same as for Studies 1 and 2. We only changed the specific instructions regarding the task participants received (“you will see two facial expressions, which will be presented one after the other with a brief delay; please focus on and categorize the emotion of the first one and ignore the second one”). Trials with neutral primes, incorrect responses (2.5%), or with response times (RTs) below 150 ms (10.2%) or above 1,500 ms (2.2%) were excluded from the main analyses.^14^ Please note that in contrast to Studies 1 and 2, we recorded response times from the onset of the prime due to the shift of focus on the prime in Study 3.

#### 4.1.1 Participants

Based on the power considerations reported for study 2, a total of 51 participants (7 men) with a mean age of 26.8 years (*SD* = 3.77 years) were recruited via the participant acquisition server at Humboldt-University. Participants were paid €8 or received course credit.

### 4.2 Results and discussion

#### 4.2.1 Reaction times

**Figure 5:**
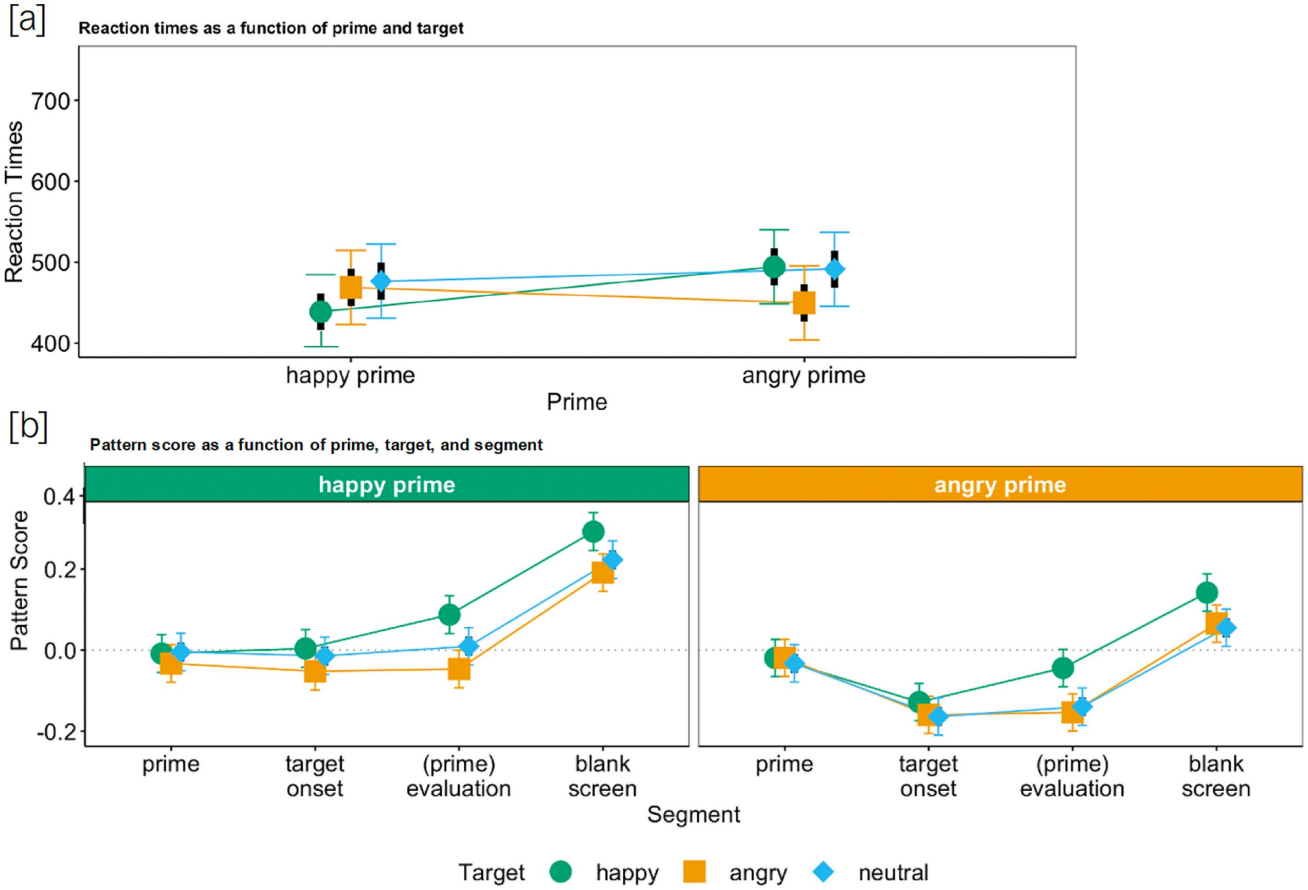
Reaction times **(a)** and pattern score **(b)** as a function of prime, target (and segment) for Study 3. Colored bars represent 95% confidence intervals and black bars represent standard errors.

As predicted, the results showed a reversal of the patterns observed in Studies 1 and 2. We found a significant main effect of prime emotion, *semi-partial R^2^* = 0.001, which however, just like in Studies 1 and 2, was qualified by an interaction between prime emotion and target emotion, *semi-partial R^2^* = 0.003: As expected (Figure 5a), the RTs were significantly shorter when prime and target emotion matched than for incongruent combinations, *b*_ang-hap_ang-hap_ = −74.3, *t* = −7.19, *p* < 0.001, *CI*_95_ ang-hap_ang-hap_ = [−94.5, −54.0] (Supplementary Table S12, which displays all coefficients of the analysis and the corresponding simple slopes), indicative of the notion that targets were processed, even though participants were told to ignore them.

#### 4.2.2 Facial muscle activity

We found that when primes are task-relevant, they are mimicked. That is, a main effect of prime emotion, *semi-partial R^2^* = 0.002, *b*_ang-hap_ = −0.10, *t* = −9.47, *p* < 0.001, *CI*_95_ang-hap_ = [−0.13, −0.08] emerged. Yet there was also a main effect of target emotion, *semi-partial R^2^* = 0.001, *b*_ang-hap_ = −0.07, *t* = −4.96, *p* < 0.001, *CI*_95_ang-hap_ = [−0.09, −0.04] without an interaction of prime and target emotion (see Figure 5B). That is, the presence of task-irrelevant targets interfered with the prime mimicry. Facial reactions in response to a happy prime were only positive when the target was also happy, *M*_hap_hap_ = 0.09, *CI*_95_ hap_hap_ = [0.07, 0.12], or when it was neutral, *M*_hap_neu_ = 0.05, *CI*_95_hap_neu_ = [0.03, 0.08], indicative of prime mimicry, but not when it was angry, *M*_hap_ang_ = 0.02, *CI*_95_hap_ang_ = [−0.01, 0.04].^15^ Similarly, facial reactions in response to an angry prime were only negative when the target was also angry, *M*_ang_ang_ = −0.07, *CI*_95_ang_ang_ = [−0.09, −0.04], or when it was neutral, *M*_ang_neu_ = −0.07, *CI*_95_ang_neu_ = [−0.09, −0.04], indicative of prime mimicry, but not when it was happy, *M*_ang_hap_ = −0.01, *CI*_95_ang_hap_ = [−0.04, 0.01] [See footnote (13)]. Consequently, we found here the reverse pattern as for Studies 1 and 2 (Figure 5b): Participants mimicked the prime but not the target reflecting the relevance of the primes to the task in Study 3, and participants’ mimicry of the prime was modulated when the target that followed the prime was incongruent in valence. Yet, in contrast to Studies 1 and 2, not only anger mimicry but happy mimicry as well was affected by an incongruent target. Since the overall pattern was strongly indicative of prime mimicry, this finding is also indicative of the notion that incongruent task-irrelevant expressions (here targets) were perceived as contextual cues that changed the meaning of the task-relevant expression (that is, the prime) and thus affected the mimicry response to that expression.

Furthermore, the time course shows that the positive change in participants’ facial responses from prime to target onset was qualified by an interaction of segment and prime emotion (see Table 5). Hence, facial responses became more negative for angry primes than for happy primes (see Table 5); that is, they did not change for happy primes and became more negative for angry primes (see Table 6). Then, from target onset to blank screen, both for angry as well as for happy primes, facial responses became more positive (see Table 5). Nevertheless, we can observe a slight increase in Corrugator S. activity (orienting response) after the appearance of the target, which was then followed by a decrease during happy primes (giving the impression that facial responses did not change for happy primes) and a further increase in Corrugator S. activity during angry primes (see Supplementary Figure S4 and Supplementary Table S13, which displays all coefficients of the analysis). Consequently, during (prime) evaluation, we can clearly observe happiness as well as anger mimicry (of the prime) when prime and target emotions matched (see Figure 5b), which, was then followed by a subtle (angry prime) or an intense (happy prime) smile in the end (after participants had made their decision about the valence of the prime). This pattern also corresponds to what was found for angry and happy targets in Studies 1 and 2.^16^

**Table 5:** Time course of participants’ facial reactions (Study 3).

Predictors	Estimates	*t*	95% CI	*p*
Segment (Target Onset–Prime)	−0.07	−4.27	[−0.10, −0.04]	**<0.001**
Segment (Target Onset–Prime) * Prime (Angry–Happy)	−0.12	−3.92	[−0.18, −0.06]	**<0.001**
Segment (Prime Evaluation– Target Onset)	0.04	2.45	[0.01, 0.07]	**0.014**
Segment (Prime Evaluation– Target Onset) * Prime (Angry–Happy)	0.001	0.02	[−0.06, 0.06]	0.98
Segment (Blank Screen–Prime Evaluation)	0.21	13.5	[0.18, 0.24]	**<0.001**
Segment (Blank Screen–Prime Evaluation) * Prime (Angry–Happy)	−0.02	−0.61	[−0.08, 0.04]	0.54

Bold values indicate *p*-values below 0.05, indicating statistical significance.

**Table 6:** Simple slopes for the interaction of segment and prime on participants’ facial reactions (Study 3).

Predictors	Estimates	*t*	95% CI	*p*
Segment (Target Onset–Prime) for Angry Prime	−0.13	−5.82	[−0.17, −0.08]	**<0.001**
Segment (Target Onset–Prime) for Happy Prime	−0.01	−0.25	[−0.05, 0.04]	0.80

Bold values indicate *p*-values below 0.05, indicating statistical significance.

*Facial reactions to neutral primes.* To address the concern that mimicry of targets occurred, even though they were deemed irrelevant in Study 3, we assessed target mimicry for the neutral primes. Similar to the argument for Studies 1 and 2, neutral primes cannot be mimicked, and thus, “mimicry” of neutral primes cannot interfere with facial muscle activity due to target mimicry. For this, we conducted an LMM analysis on facial responses to the neutral *prime*. In this analysis again, we can observe that even though facial reactions were more positive during happy targets than during angry targets, *b*_ang-hap_ = −0.06, *t* = −3.00, *p* = 0.003, *CI*_95_ang-hap_ = [−0.09, −0.02], no target mimicry occurred, as facial reactions to neutral primes were neither positive nor negative for happy targets, *M*_hap_ = −0.03, *CI*_95_hap_ = [−0.07, 0.004], and only slightly negative for angry targets, *M*_ang_ = −0.09, *CI*_95_ang_ = [−0.13, −0.05] (see Figure 3c; Supplementary Table S15, which displays all coefficients of the analysis). Consequently, participants did not mimic the target (the task-irrelevant stimulus in this study), even though targets seem to interfere with reactions to the prime for both happy and angry targets (which is not implausible given the stronger salience of the target face compared to the prime face: Whereas task relevance plays a significant role in determining emotional mimicry, the emotional content of stimuli presented immediately before or during a response cannot be entirely ignored). Overall, these findings align with Studies 1 and 2 where participants did not mimic the prime, which was the task-irrelevant stimulus in these two studies.

In sum, across all three studies, we could show that task-irrelevant faces were processed but not mimicked. They did, however, perturb mimicry responses to task-relevant faces. In Studies 1 and 2, the mimicry intensity of the angry target was reduced by the happy prime, whereas angry primes did not have an impact on happiness mimicry (see Figures 2, 4). In Study 3, angry targets delayed the mimicry of the happy prime, and happy targets also slightly reduced the mimicry intensity of the angry prime (see Figure 5). Even more importantly though, in all three studies, we found mimicry of the task-relevant face for every prime-target combination, and task-irrelevant faces were not mimicked in any of the three studies (also see Figure 3). Hence, emotional mimicry, even though thought of as a ballistic process, is susceptible to both (affective) context effects and higher-order cognitive processes such as the deliberate decision to ignore a facial expression that is deemed irrelevant for task completion. This challenges the notion that emotional stimuli always trigger automatic responses irrespective of context. Rather, as predicted by the emotional mimicry as social regular view (Hess and Fischer, 2013, 2022), emotional information is processed and responded to in line with the goals of the observer in a given context.

## 5 General discussion

The present research aimed to assess whether task-irrelevant facial expressions are mimicked. To exclude one potential explanation—that such expressions are not even processed—we used the general framework of an affective priming paradigm. In three studies we found that task-irrelevant faces were processed but not mimicked. According to our assumptions, we found that when primes were labeled as irrelevant for the rating task (Studies 1 and 2), only targets were mimicked, even when participants were told to attend to primes (Study 2). Similarly, when targets were labeled as irrelevant (Study 3), only primes were mimicked. To further illustrate that task-irrelevant expressions were not mimicked, we additionally analyzed task-irrelevant prime and neutral target combinations (Studies 1 and 2) as well as task-irrelevant target and neutral prime combinations (Study 3) and found no evidence for mimicry of task-irrelevant expressions across all three studies.

These findings are not in line with prior views on mimicry that aligned it with processes such as automatic imitation (Heyes, 2011). Specifically, automatic imitation is the automatic influence of observed movements on own voluntary movements. Even though recently some top-down influences on automatic imitation were found, automatic imitation is generally thought to make “little demand on executive function and is minimally dependent on the agent’s intentions” (Heyes, 2018, p. 501). By contrast, mimicry has been shown to be highly dependent on the meaning that is attributed to a given expression (Hess and Fischer, 2022), and part of this meaning is the *relevance* of the expression. This conclusion aligns with recent studies showing that emotional expressions and postures affect behavior only when they are relevant to the participant’s goals (Mirabella, 2018; Mancini et al., 2020, 2022; Calbi et al., 2022; Mirabella et al., 2022; Montalti and Mirabella, 2023, 2024). In these studies, emotional expressions and postures influenced motor responses only when they were central to the task. When emotions were task-irrelevant, they had no impact on behavior, emphasizing that the reaction to emotional stimuli is closely tied to their relevance within the task.

These findings support appraisal theories of emotion and the goal-directed account proposed by Moors and Fischer, which suggest that reactions to emotional stimuli are not obligatory but are instead driven by the relevance of the emotional content to the individual’s goals and objectives (Moors and Fischer, 2019; Scherer and Moors, 2019).

In conclusion, results from three well-powered studies support the notion that mimicry does not only depend on affiliation intentions of the observer (Hess and Fischer, 2013) but also on the way participants prioritize one piece of information over another. That is, individuals do not mimic task-irrelevant emotional information, even though the affective information is available. This is an important discovery, as connecting with everyone around is not always the primary goal in a social situation. In some instances, it may even hinder goal attainment. For instance, consider a situation where you are discussing a personal problem with a friend in a café, and you notice people around you laughing and smiling. It would be considered impolite to automatically mimic these “irrelevant” background expressions during your conversation. Another scenario could be a business setting where you discuss a serious project with some colleagues, while other colleagues speak amongst themselves. Even though the latter might be displaying cheerful expressions, it would be counterproductive and inappropriate for you to mimic those emotions, as it could undermine the gravity of the discussion. Hence, if two possible mimicry targets are presented (as in our present design), our findings suggest that the more “relevant” target is mimicked. Thus, it seems that observers prioritize relevant emotional cues over those that are contextually present but deemed irrelevant. Doing so can aid in maintaining appropriate emotional boundaries and effectively navigating complex social situations.

Nonetheless, in all three studies, incongruent irrelevant facial expressions influenced the level (but not the occurrence) of mimicry of relevant facial expressions. Emotional expressions that precede (or follow) other emotional expressions or that are shown in the background but are deemed irrelevant for task completion may represent context cues that can modulate judgments about others in terms of their likability and affiliativeness (Ferguson et al., 2005; Anderson et al., 2012), which then influence own facial expression responses (Katembu et al., 2022). Importantly, in our studies the identity of the person showing the prime and the target expressions was always the same. Hence the preceding (Studies 1 and 2) or following (Study 3) task-irrelevant expressions were still informative about the person that showed the task-relevant expression.

The effect of facial context on mimicry seems to depend on valence. Similar to Philip et al. (2018), we found that task-irrelevant positive contexts had stronger effects on mimicry than task-irrelevant negative contexts. This leads to the alternative explanation that the presentation of a happy facial prime induced a corresponding affective state, as affect may be “a genuine online component of perception” (Topolinski et al., 2015). This is indeed more likely to happen for happy than for angry faces (also see, e.g., Hess and Blairy, 2001; Wróbel and Olszanowski, 2019). This positive affective state induced by the happy facial prime then may have interfered with the negative facial mimicry reaction. In contrast, mimicry of happy facial expressions was less context-dependent and was only influenced by the angry target in Study 3. In general, compared to anger mimicry, happy mimicry is a more robust phenomenon that is less affected by top-down information (Hess, 2021).

An alternative explanation is that happy facial expressions were more arousing than corresponding angry facial expressions, making them a stronger context as well as a stronger target. Our results show that happy expressions were detected faster; thus, they probably conveyed stronger emotional arousal than their angry counterparts (Lundqvist et al., 2014), This means that happy contexts and targets were probably more prominent and influential in shaping participants’ perceptions and (mimicry) responses, as they tended to capture and hold participants’ attention more effectively.

In contrast to Studies 1 and 2, in Study 3 mimicry to both angry and happy primes was affected by an incongruent target. This effect can be attributed to the target face’s stronger salience compared to the prime face, also evident in the reaction times for the prime ratings in Study 3. Hence, the influence of the context seems to have been stronger in Study 3 compared to Studies 1 and 2. Nonetheless, individuals were very accurate in their judgments in Study 3 as well.

Another interesting finding in Study 3 was that the evaluation of a face was not only influenced by preceding faces but also by faces that were displayed *after* the relevant face. This essentially is backward affective priming (Fockenberg et al., 2006), for which empirical evidence is scarce and up to date limited to studies with words as primes. Similarly, as noted above, we also found context effects on mimicry in Study 3, even though the context followed the task-relevant expressions. Thus, the evaluation of and the reaction to facial displays is a dynamic process that does not stop with the end of the task-relevant face. On the contrary, contextual cues such as task-irrelevant emotional faces—even if they follow the task-relevant face—influence the speed with which a task-relevant face is evaluated as well as empathic responses to that face. This is an important finding, as during social interactions we do not only see and possibly react to other individuals prior to starting a conversation with one specific individual but “irrelevant” others will also walk by and look at us displaying different emotions after we have already started a conversation. It is therefore just as relevant to study whether a second stimulus influences judgments and the nonverbal reactions to a previous one than to study the inverse effects.

Our results suggest that incongruent task-irrelevant faces interfere with mimicry responses to task-relevant faces. We interpret this as an interference effect with incongruent task-irrelevant faces changing the interpretive context for task-relevant faces. However, interference may not stem solely from shifts in task relevance itself but also from the cognitive demands of managing competing emotional cues. When participants encounter task-relevant and task-irrelevant expressions displaying opposing emotions, they face conflicting demands on their attention. This attentional conflict diverts focus from the primary task, making it harder to concentrate solely on the task-relevant expression. The cognitive system has to allocate resources to manage this “competition” for attention, which slows processing (i.e., affective priming effect) and may then also reduce the intensity of mimicry toward the task-relevant expression. This explanation, however, does not explain why happy faces produce stronger interference effects than angry faces, especially given that anger from an evolutionary standpoint should capture attention more readily.

### 5.1 Strengths and limitations

The present research provides important insights into the type of factors that influence emotional mimicry. In accordance with our assumptions, we found that emotional mimicry is not solely influenced by affective factors, like the affective stance toward an interaction partner, but also by cognitive factors, such as the relevance of emotional expressions for task completion. That is, across all three studies, mimicry occurred exclusively in response to task-relevant faces even though task-irrelevant faces were processed. The strong coherence of findings across the three different studies with exactly the same design and only slightly differing instructions suggests that the mechanisms revealed here are fundamental and valid for a wide range of different situations. Still, our research has also limitations.

First, we included neutral primes and targets in our design to be able to investigate whether task-irrelevant faces are mimicked or not. Our findings confirm the assumption that this is indeed not the case. Including other types of primes (and targets), however, is a major change in the affective priming design, which could have influenced the evaluation of targets in Studies 1 and 2 (and primes in Study 3). Yet, the response time advantage of congruent trials (over incongruent trials) was evident in all three studies and neutral task-irrelevant faces seemed to have played a similar role as incongruent task-irrelevant faces. This is in line with findings by Hermans et al. (1994) who also included neutral primes to be able to evaluate whether interference or facilitation or both explains affective priming effects.

Additionally, we did not measure the arousal levels of the facial expressions used, and the Radboud database lacks arousal ratings. Arousal is known to play a significant role in the speed at which emotions are detected (Lundqvist et al., 2014). Consequently, it is possible that, as mentioned above, happy expressions might have served as a stronger stimulus compared to angry expressions, leading to an imbalance in the incongruent pairs (i.e., happy prime and angry target or angry prime and happy target). However, since our focus was not on the differential effects of specific emotions as targets or primes, but rather on the overall difference between task-relevant and task-irrelevant stimuli, this limitation should not have significantly impacted the main conclusions of our study.

Further, there was only a short period of time for the assessment of mimicry, because in an affective priming design participants are asked to react as fast as possible to the stimulus of relevance and these reactions typically do not take longer than 500–700 ms (and definitely not longer than 1,500 ms, which is often used as upper limit in the RTs’ outlier analysis, see, e.g., Hermans et al., 2001). Upon task completion participants started smiling, overriding any mimicry reactions. This differs notably from the classical “mimicry task” where participants see facial stimuli for a period of at least 4–6 s (e.g., Mauersberger et al., 2015). It could be argued that this restricted temporal window might have curtailed the depth and intensity of the mimicry response. However, the onset of anger and happy mimicry usually occurs within 300–500 ms after stimulus onset and peaks at 1000 ms (Dimberg et al., 2002). As such, there was enough time for mimicry to emerge. This is supported by the fact that we consistently identified clear patterns of happiness and anger mimicry across all three studies. The robust and consistent nature of these mimicry effects within the compressed time frame raises the possibility that our methodology could have leaned toward a rather conservative stance in addressing whether only task-relevant faces trigger mimicry. In fact, it is plausible that an extended time frame might have yielded even more pronounced and prolonged mimicry responses of task-relevant faces. Nevertheless, for future investigations, alternative paradigms could be explored to address potential temporal constraints. Given the lack of studies on the impact of individual differences and contextual factors on the time course of mimicry responses, this would be an interesting avenue for future research.

A related problem is that there are only 200 ms between prime end and target onset. This allows for the argument that prime mimicry occurred and then was aborted and overwritten by target mimicry. However, if this had been the case, prime mimicry should have been observed in the neutral target condition in Studies 1 and 2, as in this condition no stimulus able to override an existing mimicry response was presented.^17^ However, no evidence of prime mimicry was found in the neutral target condition, neither in Study 1 nor 2. Further, mimicry has been shown to be a ballistic process that once started it cannot be stopped (Dimberg et al., 2002); therefore, mimicry is unlikely to be aborted by top-down processes once it has started.

Another potential limitation is that individual traits such as empathy or emotional intelligence, which may influence attention processes and emotional mimicry, were not measured or controlled for. Yet, even though these traits may affect emotional processing more broadly, they are unlikely to systematically impact our findings, as our studies employed a within-subjects design where responses to different conditions are compared within each participant. Nonetheless, future studies may consider including measures of individual differences as covariates to explore whether or not they influence mimicry of task-relevant and task-irrelevant emotional expressions.

Finally, none of the studies achieved a balanced sample in terms of gender distribution. The practical constraints encountered—such as participant availability, recruitment challenges and limitations in resources—rendered this unfeasible. However, given that the influence of gender on the recognition of basic facial expressions of emotion remains inconclusive (Forni-Santos and Osório, 2015; Mezentseva et al., 2022), and, even more importantly, since the primary objectives of our studies did not include a comparison of male and female observers, we believe this imbalance does not significantly impact the validity of our findings.

### 5.2 Conclusion

Emotional mimicry is an important facet of social interaction quality, as it fosters mutual satisfaction and liking. We examined whether emotional mimicry may be influenced by the deliberate decision to focus on other aspects of a situation while watching a person’s emotional facial expressions. In three studies we found strong support for the hypothesis that task-irrelevant faces are not mimicked, even though the expressions shown were processed, supporting the notion that emotional information is processed and responded to in alignment with the observer’s goals in a given context (Hess and Fischer, 2013, 2022).

## Data Availability

The datasets presented in this study can be found in online repositories. The names of the repository/repositories and accession number(s) can be found at: http://doi.org/10.17605/OSF.IO/R3VK4.

## Footnotes

^1^It should be noted that mimicry can be observed during passive viewing. It is however plausible that participants take note of emotional expressions during that task.

^2^The Corrugator S. is a facial muscle that is located between the eyebrows; its activation creates vertical furrows or wrinkles between the eyebrows resulting in a frown.

^3^The Zygomaticus M. is a facial muscle that is primarily responsible for elevating the corners of the mouth; its activation creates a smile expression (in combination with a relaxation of the Corrugator S.).

^4^The debriefing included a clear explanation of the study’s objectives, during which participants were informed about the research’s goals, methodologies, and implications, and were encouraged to share their thoughts and ideas, thereby upholding ethical practices, participant wellbeing, and fostering an open dialog between researchers and participants.

^5^We chose actors with a high recognition rate for happy and angry faces (> 85%).

^6^The distribution of errors for each combination of prime and target can be found in Supplementary Table S1.

^7^We used the second prior to the presentation of the prime as baseline for each trial.

^8^LMMs offer distinct advantages over traditional repeated-measures ANOVAs for analyzing experimental data (where participants respond to multiple trials). ANOVAs have limitations such as the loss of variability information. LMMs are capable of taking into account dependencies, continuous predictors, and various response variables; hence they yield better interpretability and fit to the data compared to traditional methods (Brown, 2021).

^9^*Semi-partial R^2^* is an effect size measure for fixed effects in LMMs, which was calculated following Nakagawa and Schielzeth (Jaeger et al., 2017).

^10^Similarly to Cannon et al. (2009), happy targets were easier to detect and thus were generally detected faster than angry targets (see the RTs when neutral primes preceded targets). Thus, to unconfound the priming effect from this difference in speed, we compared the differences between the primes separately for happy and angry targets: The RTs to both angry and happy targets were significantly shorter when the preceding prime was congruent than incongruent, simple slope z_ang_ang-hap_ = − 32.0, t = − 7.23, *p* < 0.001, CI_95_ang_ang-hap_ = [− 40.7, − 23.3], z_hap_hap-ang_ = − 45.0, t = − 10.2, *p* < 0.001, CI_95_hap_hap-ang_ = [− 53.7, − 36.4].

^11^Here as well the distribution of errors for each combination of prime and target can be found in the Supplementary Table S6.

^12^In Study 2 again, happy targets could be detected easier and thus were generally detected faster than angry targets (see the RTs when neutral primes preceded targets), Thus, to unconfound the priming effect from this difference in speed, we compared the differences between the primes for happy and angry targets: The RTs to both angry and happy targets were significantly shorter when the preceding prime was congruent than incongruent, simple slope z_ang_ang-hap_ = − 36.1, t = − 5.59, *p* < 0.001, CI_95_ang_ang-hap_ = [− 48.8, − 23.5], z_hap_hap-ang_ = − 47.9, t = − 7.44, *p* < 0.001, CI_95_hap_hap-ang_ = [− 60.5, − 35.3].

^13^In backward affective priming, the temporal order of the relevant stimulus and the irrelevant stimulus are reversed: the irrelevant stimulus is presented *after* the relevant stimulus. Despite this reversed presentation sequence, the emotional valence of the irrelevant stimulus still influences the processing of the relevant stimulus. The concept of backward affective priming challenges the traditional assumption that the emotional influence flows only from one stimulus to another in a forward direction.

^14^The distribution of errors for each combination of target and prime can be found in the Supplementary Table S11.

^15^Please note that here the confidence interval is not different from zero, which means that even though there was no prime mimicry, there was also no target mimicry.

^16^In all three studies, we also examined whether emotion recognition accuracy and reaction times related to mimicry behaviors (see Supplementary Figures S4, S9, S14). We only found this relationship in Study 2 where longer reaction times were associated with an increase in the emotional mimicry response. This might be explained by the increased interest in targets in these situations: The longer it takes to decide about the valence of the target, the more one is interested in the target and hence the more intensely one mimics it. Yet, it is important to note that this is not a consistent finding across studies. In Studies 1 and 3, we found a more or less negative effect of reaction times on positive facial reactions. Slower reactions were associated with positive facial reactions independent of the target’s expression (Study 1) and for happy primes (Study 3). This may reflect concentration processes in situations of uncertainty. This also fits to the negative effect of accuracy on positive facial reactions in Study 1. In situations where participants concentrated more, they were more accurate than in situations where they did not concentrate that much.

^17^Neutral expressions might convey subtle affective cues depending on the morphology of actors’ faces (Adams et al., 2012). Yet, we used multiple actors with different facial features to reduce the likelihood of systematic biases.
