# Supplementary material for: Task-irrelevant emotional expressions are not mimicked, but may modulate the mimicry of task-relevant emotional expressions
Source: Front Psychol. 2025 Jan 7;15:1491832. doi: 10.3389/fpsyg.2024.1491832 (PMC11748183; doi:10.3389/fpsyg.2024.1491832)
Supplement: Supplementary file 1 [file Data_Sheet_1.docx]

Supplementary Material

# Supplementary Figures and Tables

## Supplementary Tables

Table S1. Distribution of errors (Study 1)

| *Prime* | *Happy Target* | *Angry Target* |
| --- | --- | --- |
| Happy | 159 | 130 |
| Angry | 143 | 145 |
| Neutral | 130 | 119 |

Table S2. Reaction times as a function of target and prime (Study 1)


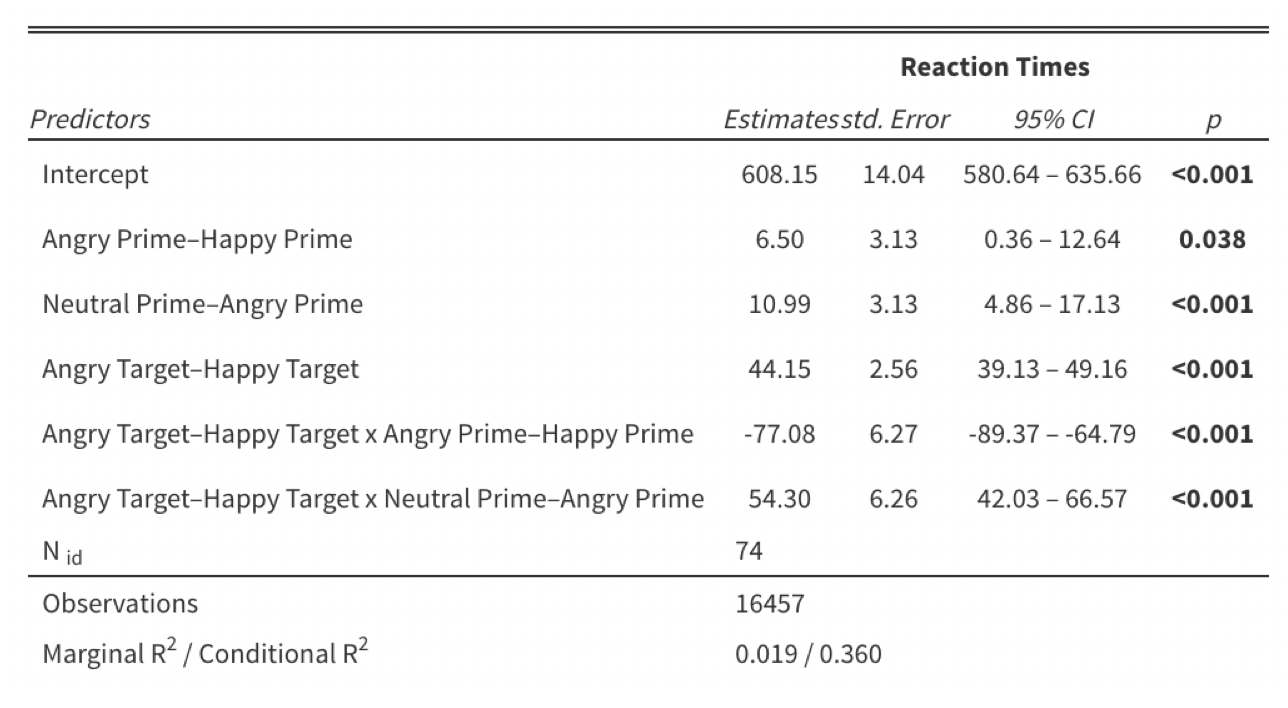


*Note.* When neutral primes preceded targets, happy targets were identified faster than angry targets, simple slope *z*_ang-hap_neu_ = 54.7, *t* = 12.4, *p* < .001, *CI*_95_ang-hap_neu_ = [46.0, 63.3] and this difference was even larger when happy primes preceded targets, simple slope *z*_ang-hap_hap_ = 77.4, *t* = 17.5, *p* < .001, *CI*_95_ang-hap_hap_ = [68.7, 86.1], but the faster reaction to happy targets was not found when angry primes preceded targets, simple slope *z*_ang-hap_ang_ = 0.36, *t* = 0.08, *p* = .94, *CI*_95_ang-hap_ang_ = [- 8.33, 9.04].

Table S3. Participants’ facial reactions as a function of target, prime and segment (Study 1)


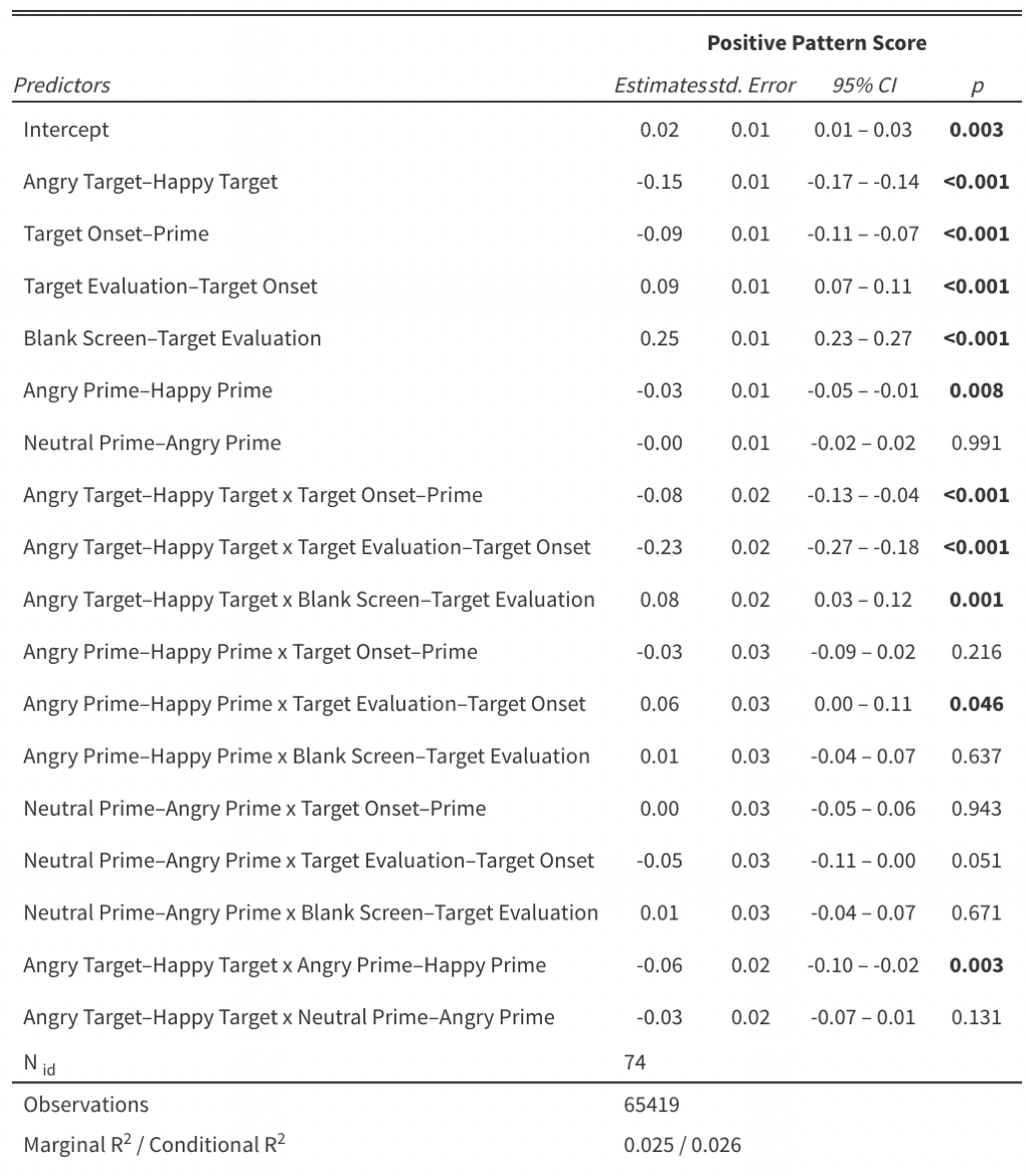


Table S4. Participants’ facial reactions as a function of hit rate and reaction times (Study 1)


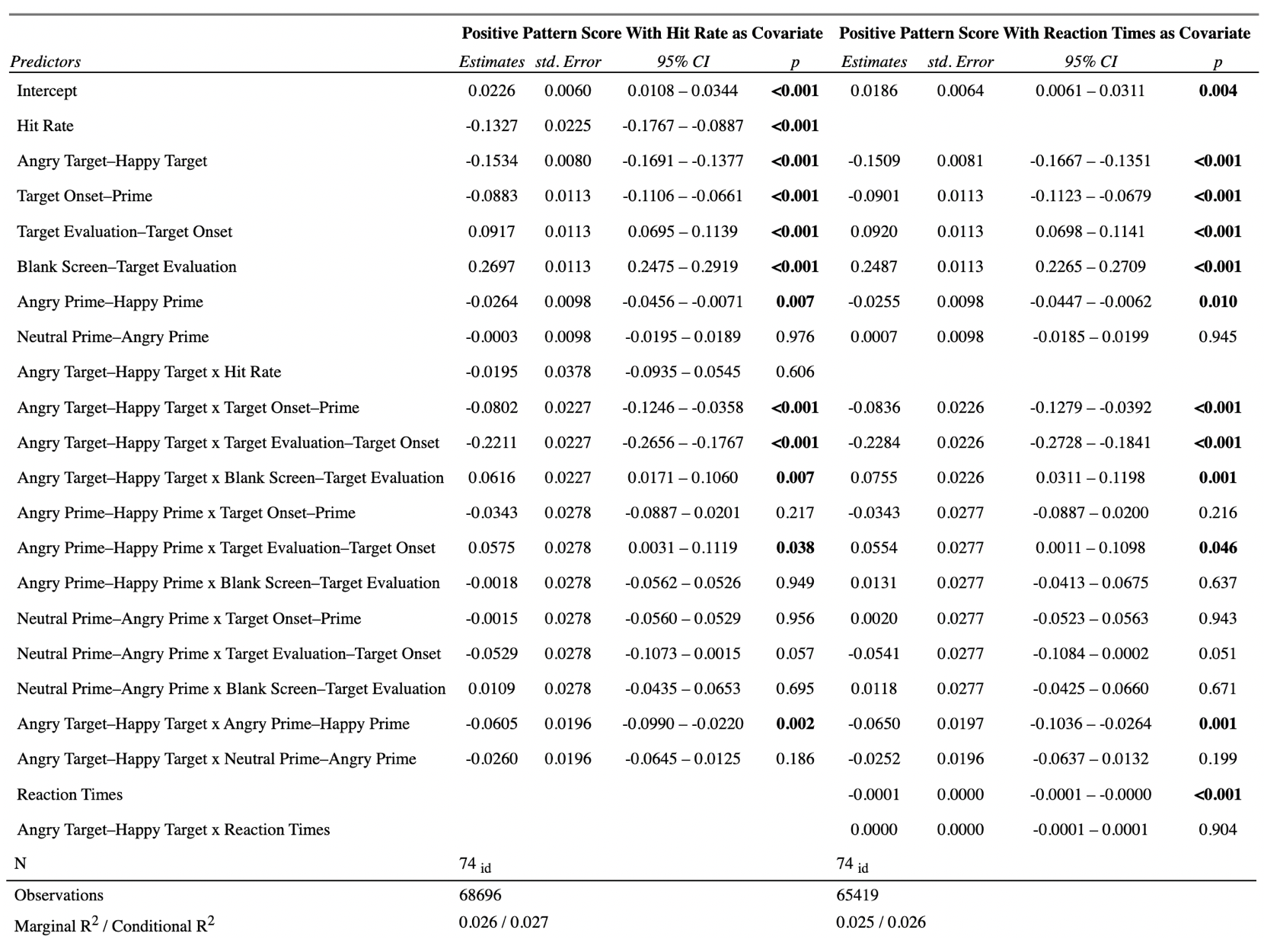


Table S5. Participants’ facial reactions in the “neutral target”-condition (Study 1)


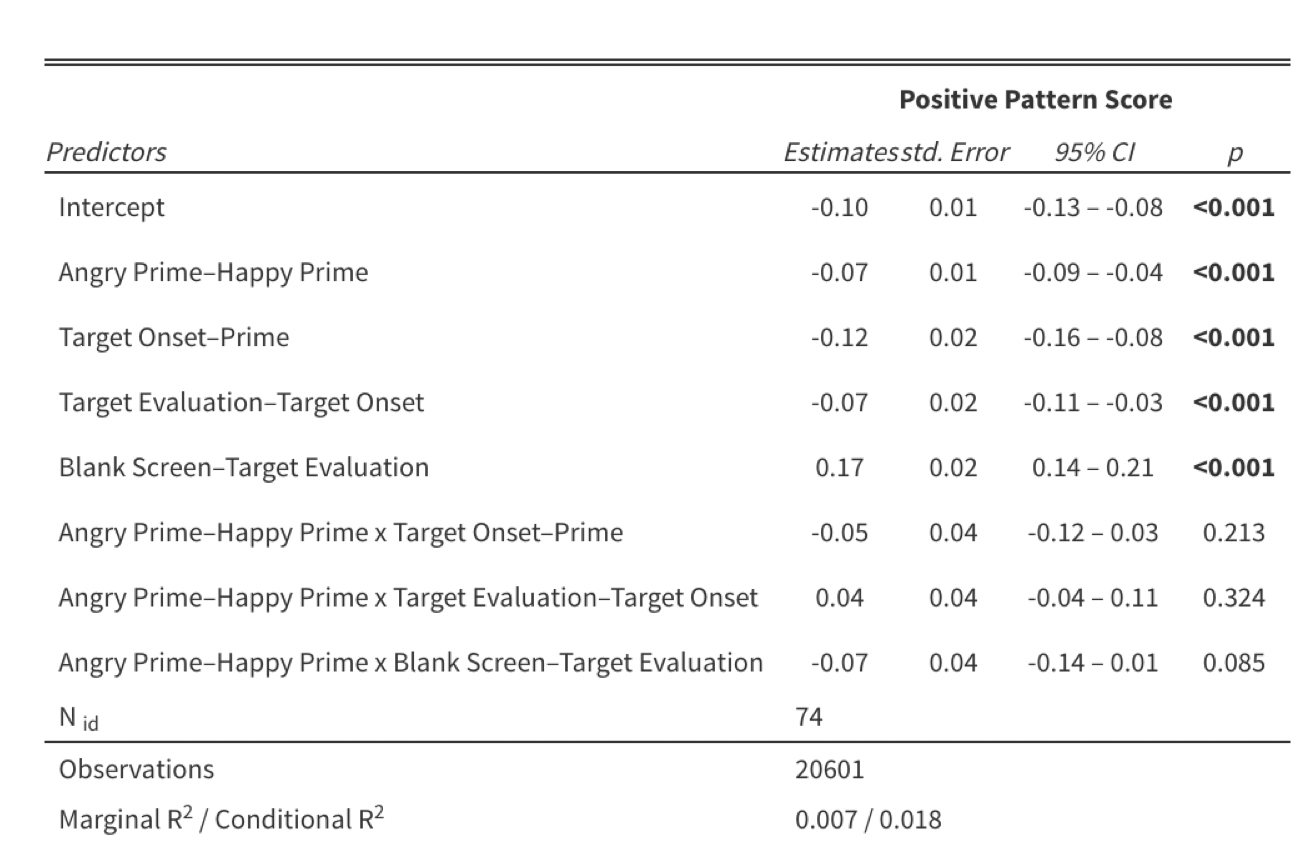


Table S6. Distribution of errors (Study 2)

| *Prime* | *Happy Target* | *Angry Target* |
| --- | --- | --- |
| Happy | 40 | 111 |
| Angry | 143 | 36 |
| Neutral | 92 | 63 |

Table S7. Reaction times as a function of target and prime (Study 2)


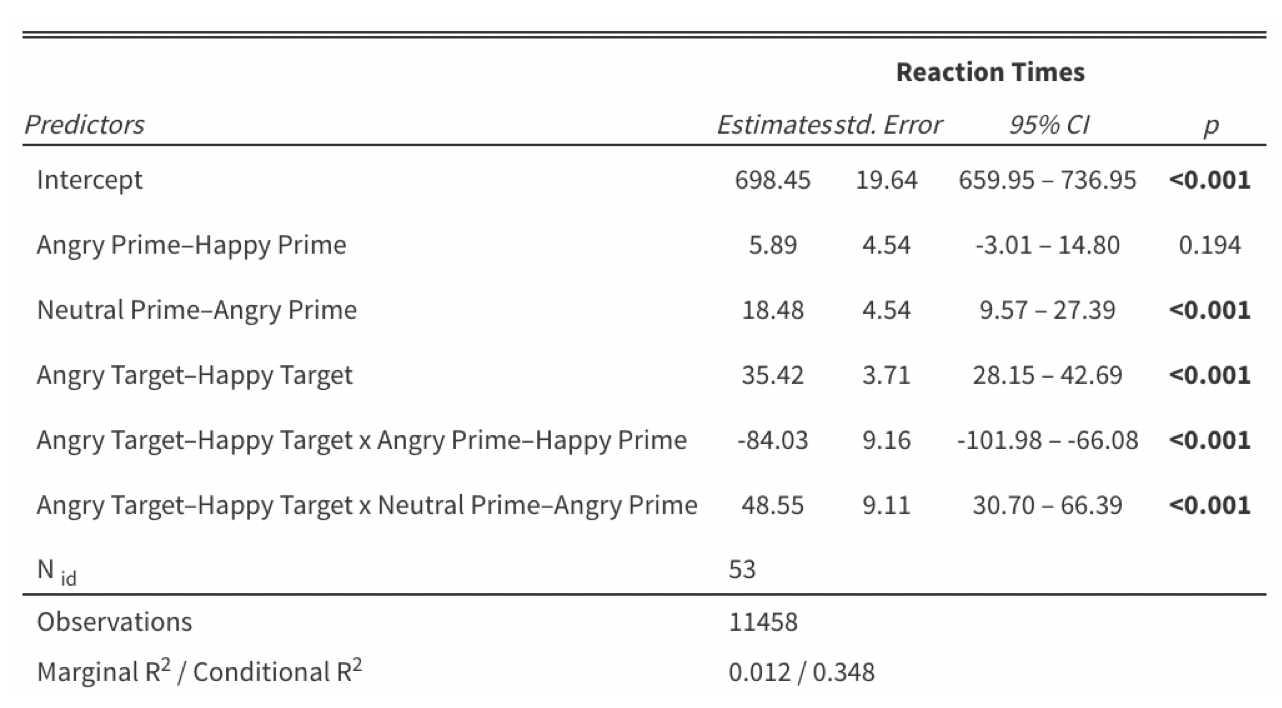


*Note*. When neutral primes preceded targets, happy targets were identified faster than angry targets, simple slope *z*_ang-hap_neu_ = 39.8, *t* = 6.19, *p* < .001, *CI*_95_ang-hap_neu_ = [27.2, 52.4], and this difference was even larger when happy primes preceded targets, simple slope *z*_ang-hap_hap_ = 75.3, *t* = 11.7, *p* < .001, *CI*_95_ang-hap_hap_ = [62.6, 87.9], but the faster reaction to happy targets was not found anymore when angry primes preceded targets, simple slope *z*_ang-hap_ang_ = -8.77, *t* = - 1.36, *p* = .17, *CI*_95_ang-hap_ang_ = [- 21.4, 3.90].

Table S8. Participants’ facial reactions as a function of target, prime and segment (Study 2)


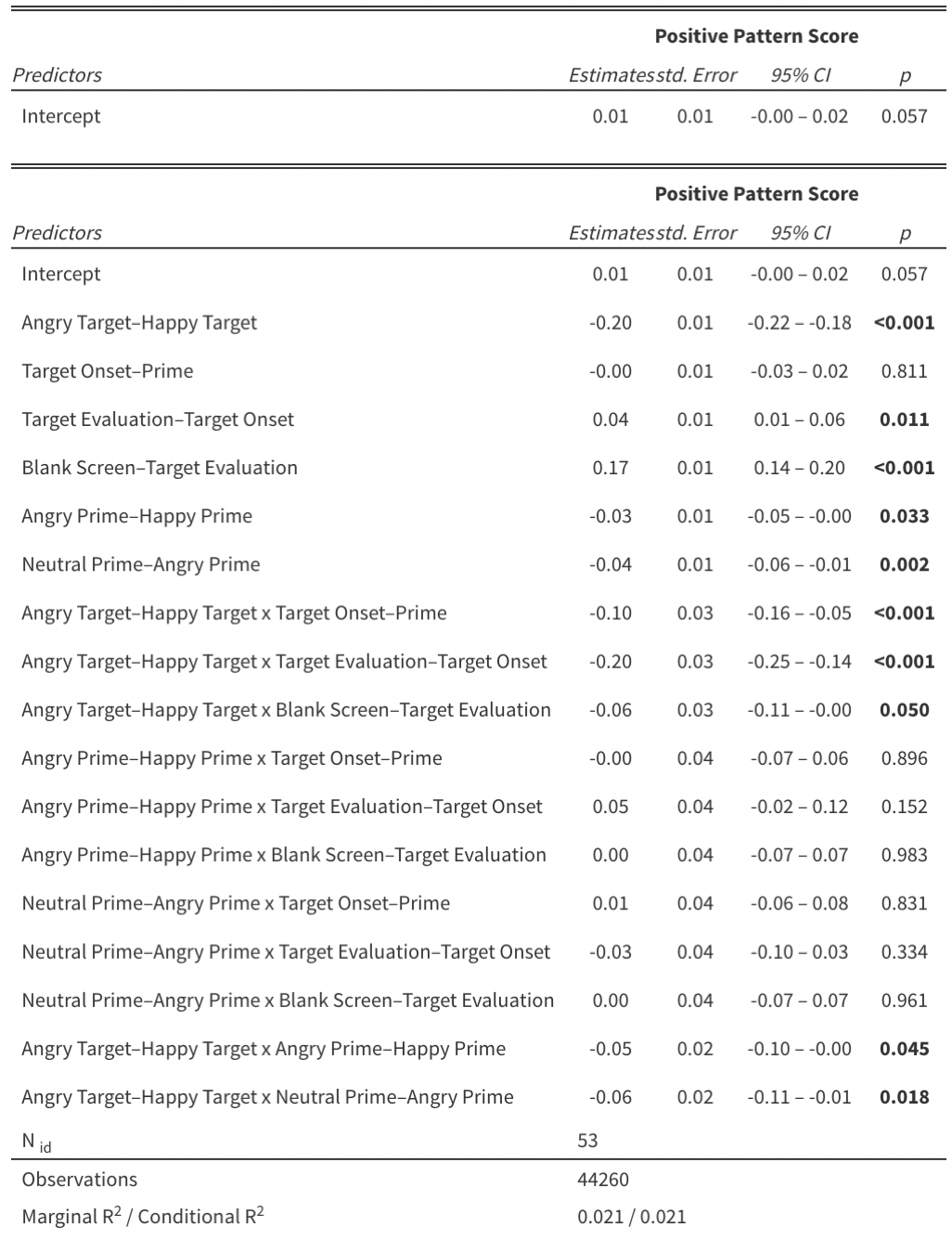


Table S9. Participants’ facial reactions as a function of hit rate and reaction times (Study 2)


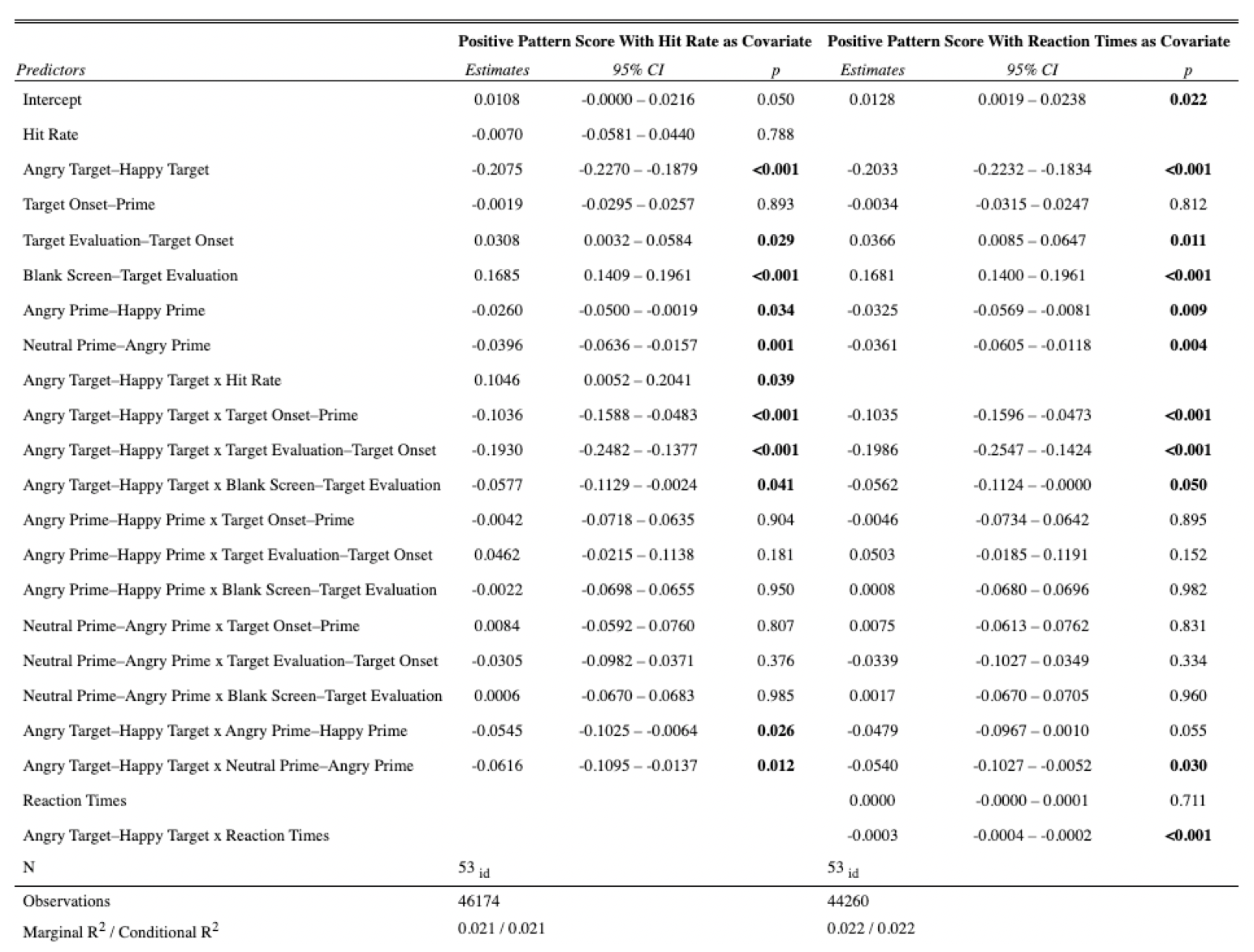


Table S10. Participants’ facial reactions in the “neutral target”-condition (Study 2)


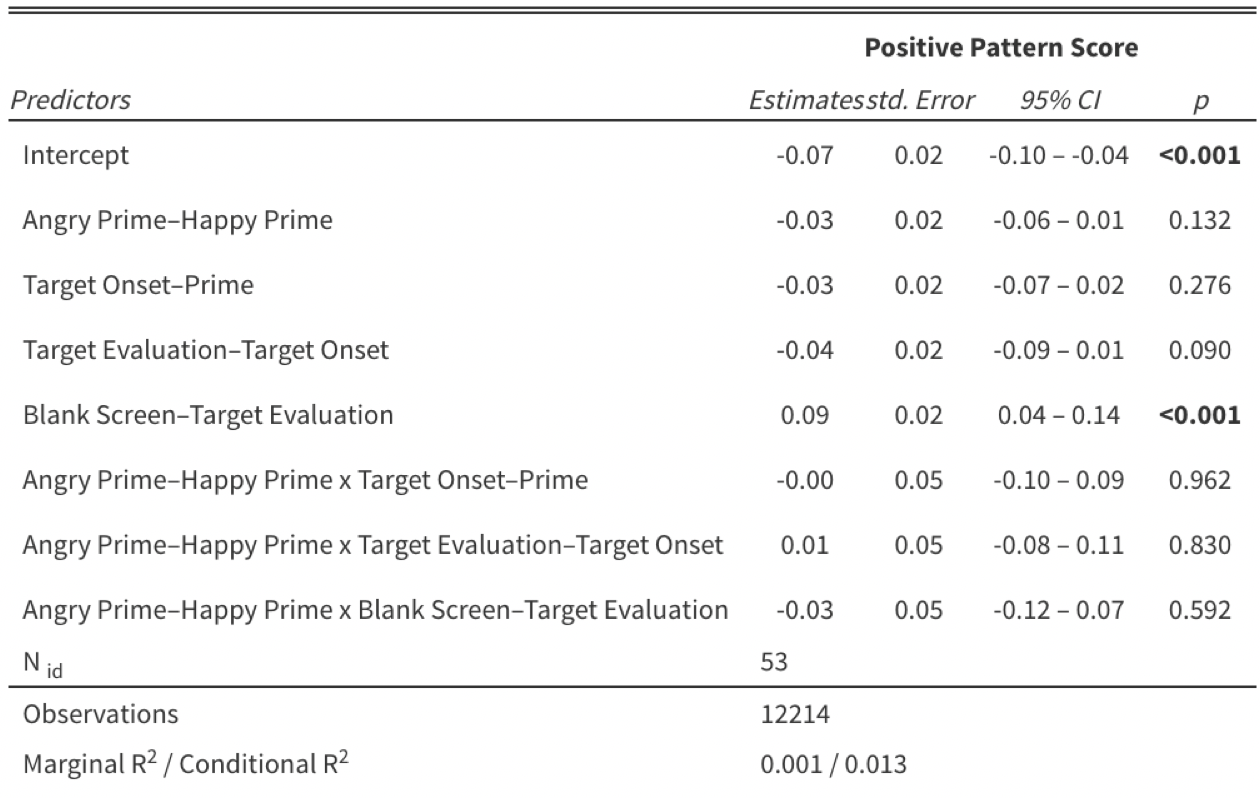


Table S11. Distribution of errors (Study 3)

| *Target* | *Happy Prime* | *Angry Prime* |
| --- | --- | --- |
| Happy | 44 | 46 |
| Angry | 59 | 25 |
| Neutral | 55 | 39 |

Table S12. Reaction times as a function of target and prime (Study 3)


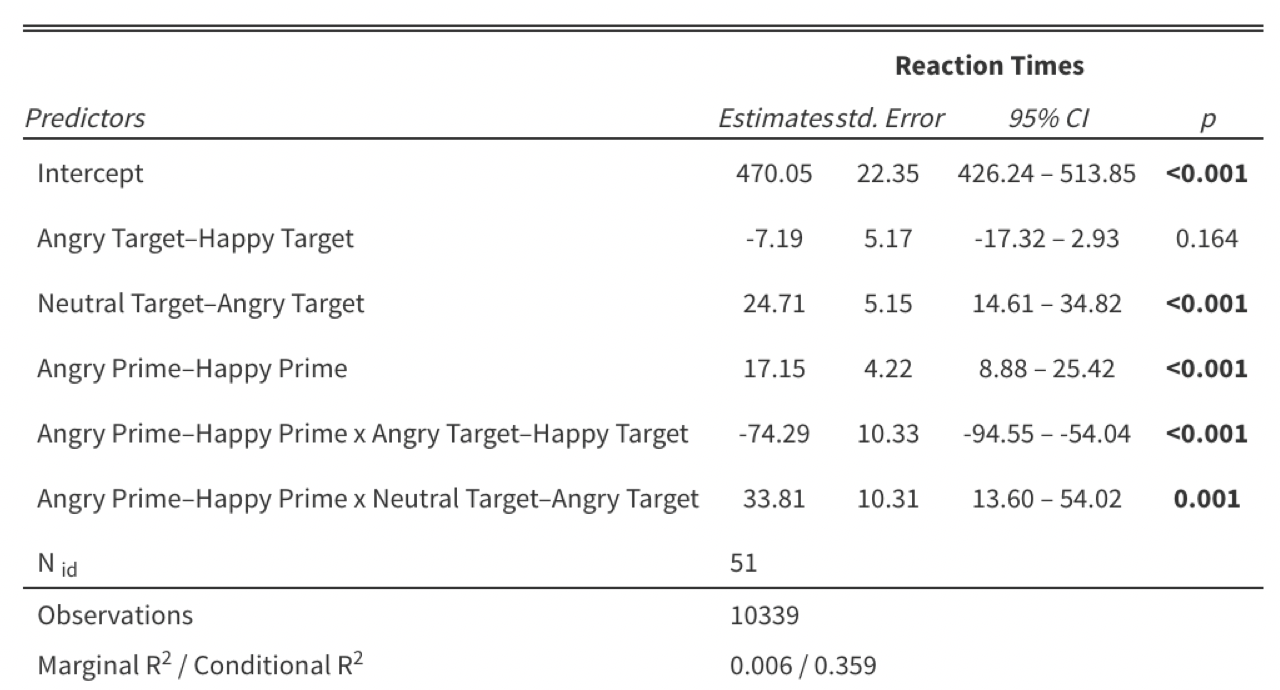


*Note*. Happy primes were only identified slightly faster than angry primes when neutral targets followed primes, simple slope *z*_ang-hap_neu_ = 14.9, *t* = 2.05, *p* = .041, *CI*_95_ang-hap_neu_ = [- 0.63, 29.2]. In contrast, happy primes were identified substantially faster than angry primes when happy targets followed primes, simple slope *z*_ang-hap_hap_ = 55.4, *t* = 7.56, *p* < .001, *CI*_95_ang-hap_hap_ = [41.1, 69.9] and angry primes were identified faster than happy primes when angry targets followed primes, simple slope *z*_ang-hap_ang_ = - 18.9, *t* = - 2.59, *p* = .010, *CI*_95_ang-hap_ang_ = [- 33.2, - 4.60].

Table S13. Participants’ facial reactions as a function of target, prime and segment (Study 3)


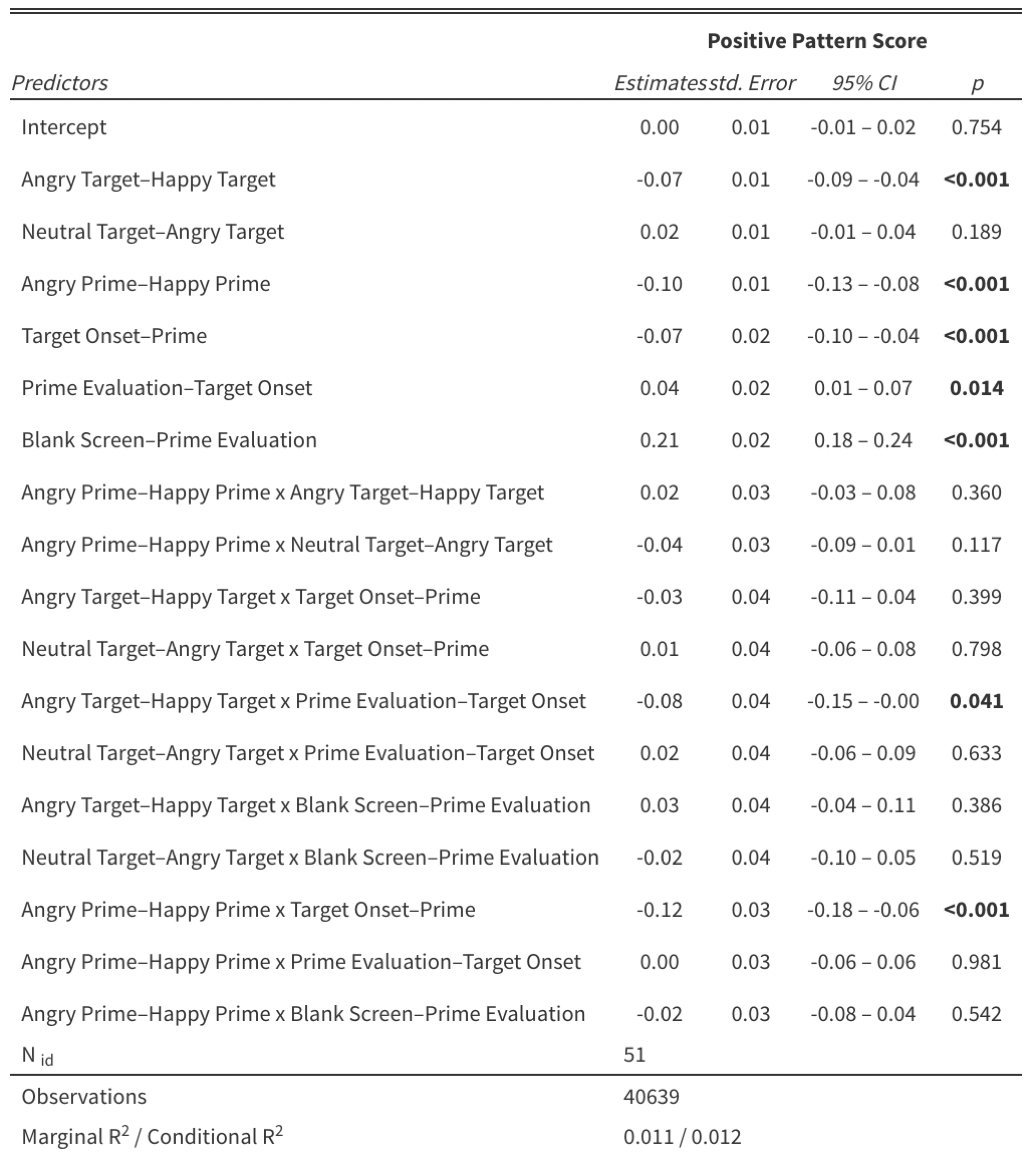


Table S14. Participants’ facial reactions as a function of hit rate and reaction times (Study 3)


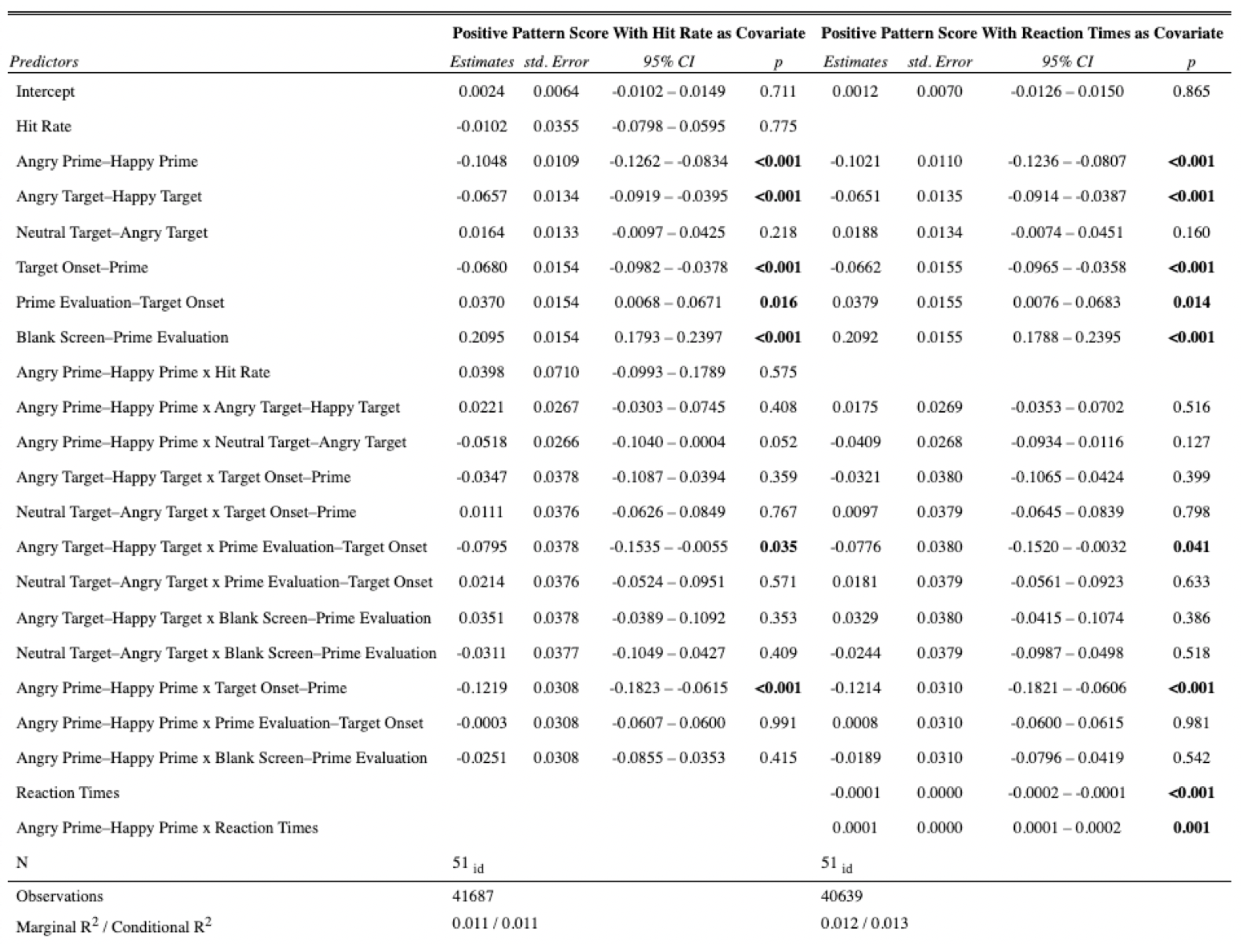


Table S15. Participants’ facial reactions in the “neutral prime”-condition (Study 3)


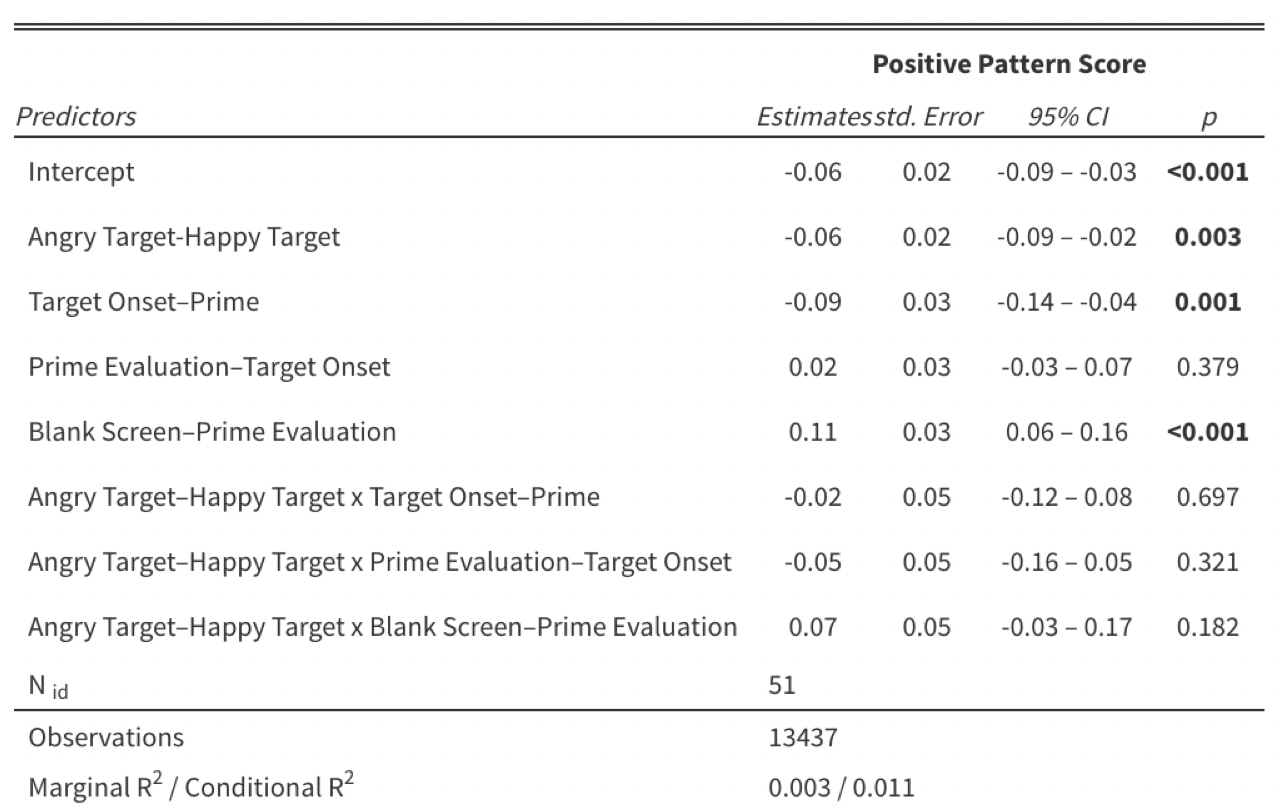


## Supplementary Figures


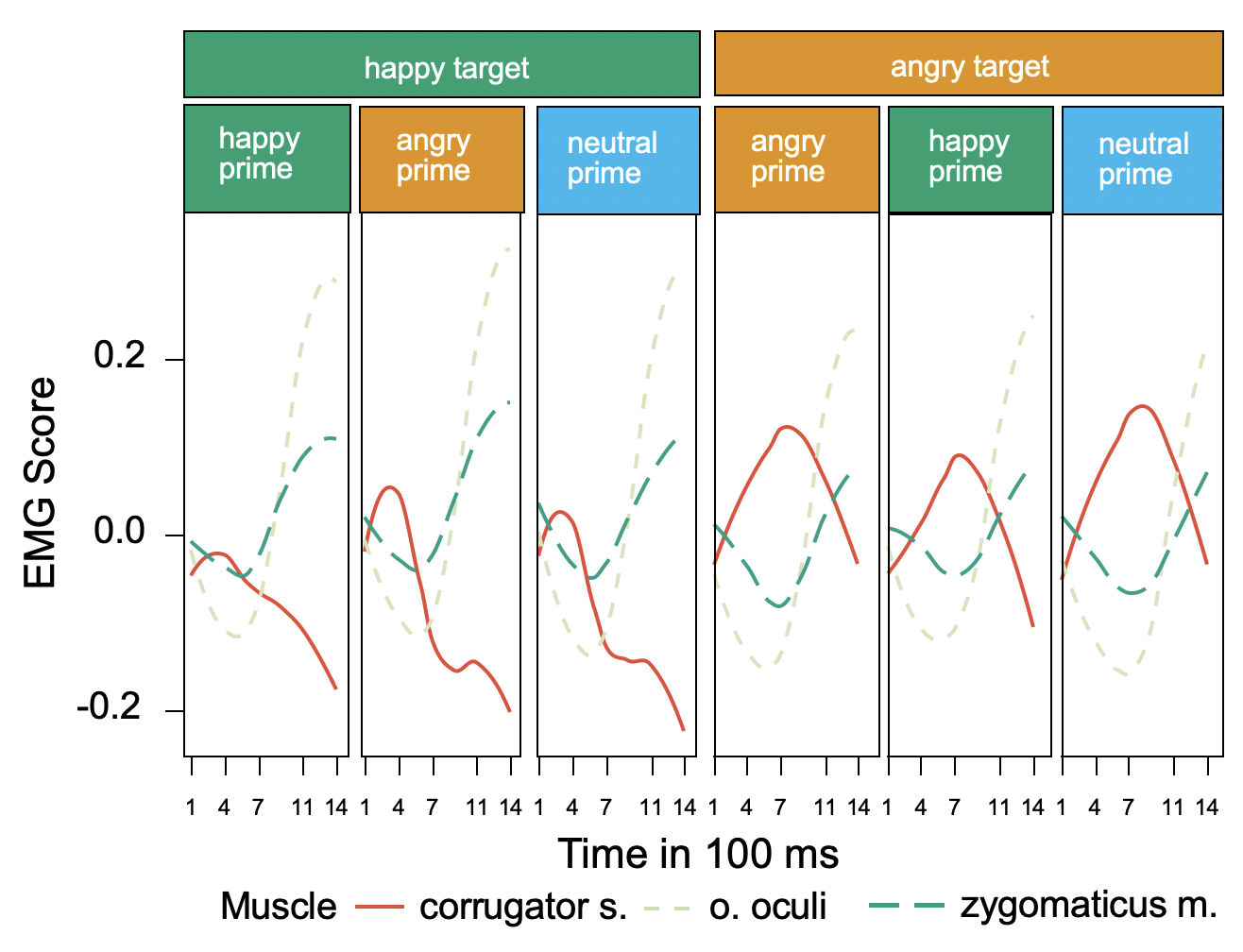


**Figure S1.** Muscle activity as a function of target, prime, muscle and time (Study 1)


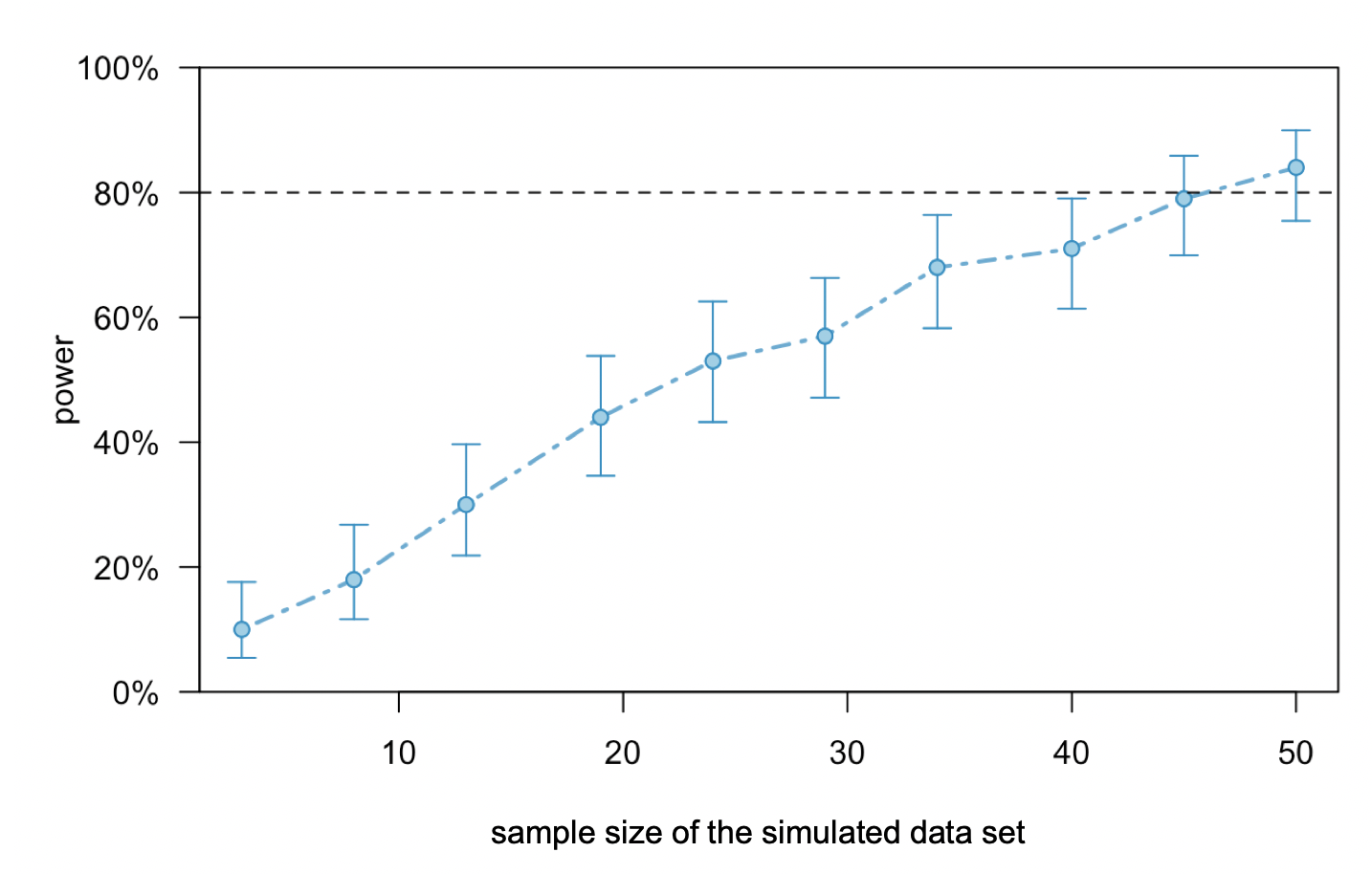


**Figure S2.** Power curve to estimate the minimum sample size based on data from Study 1.


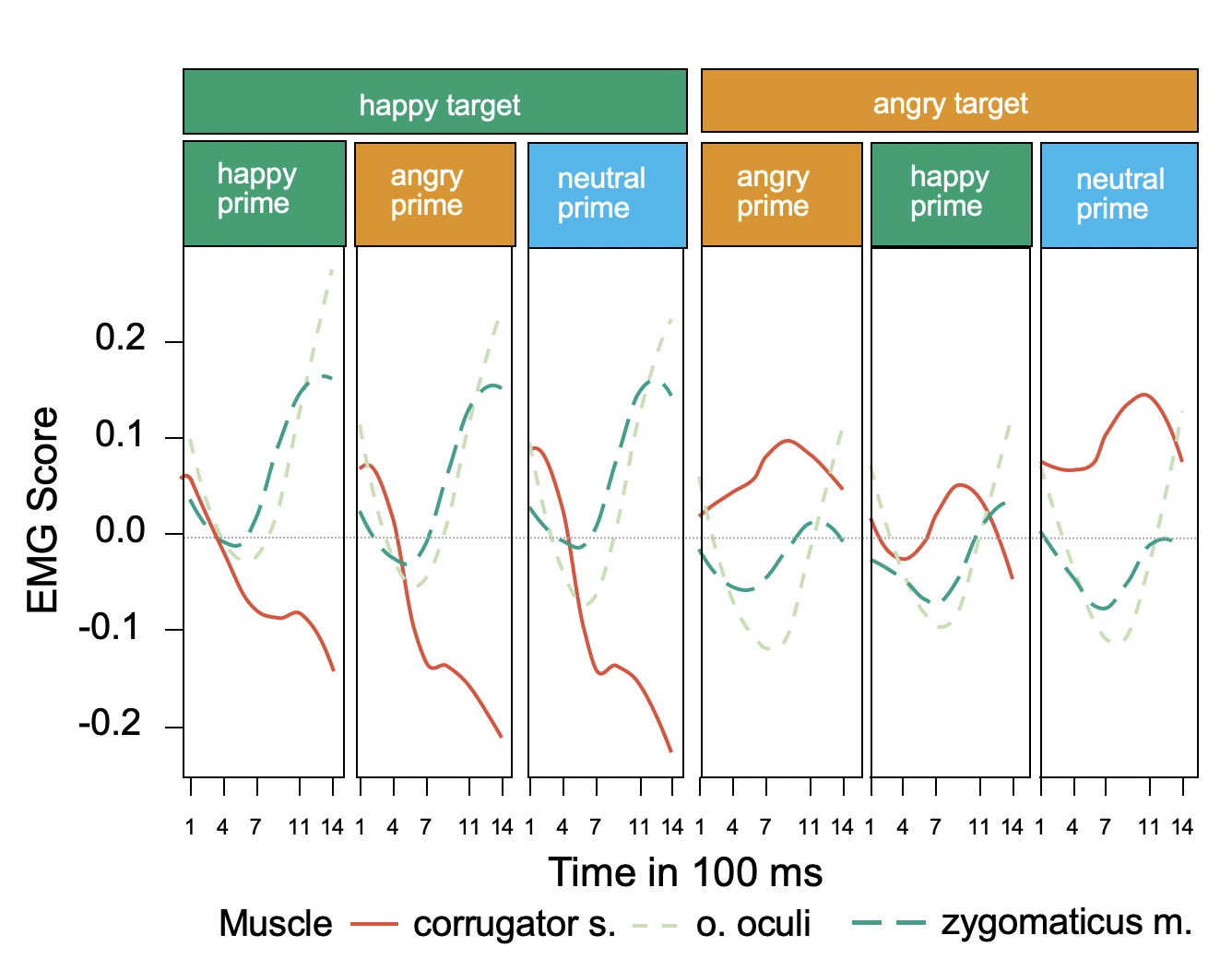


**Figure S3.** Muscle activity as a function of target, prime, muscle and time (Study 2)


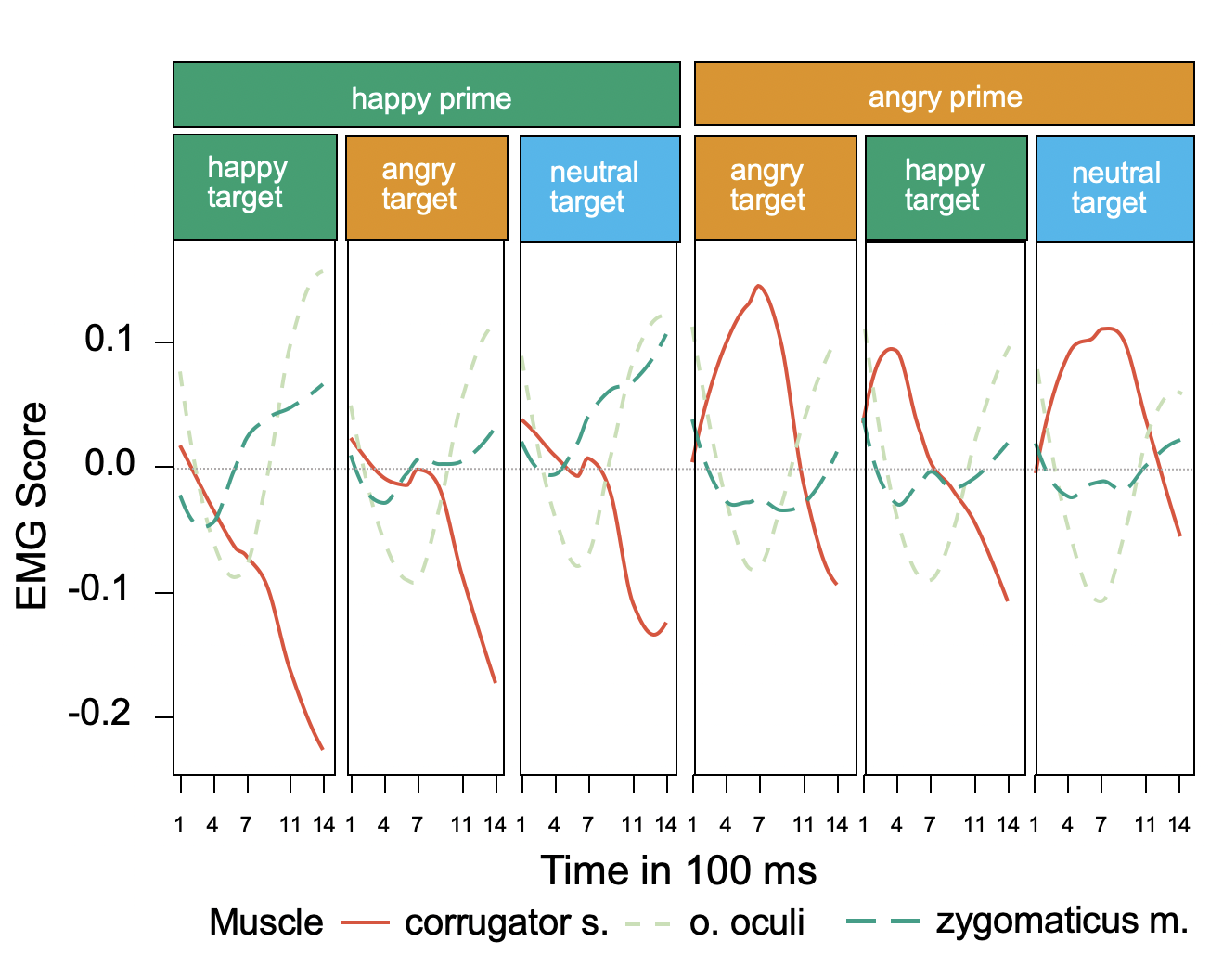


**Figure S4.** Muscle activity as a function of prime, target, muscle and time (Study 3)
